# Genome-based taxonomic rearrangement of *Oceanobacter*-related bacteria including the description of *Thalassolituus hydrocarbonoclasticus* sp. nov. and *Thalassolituus pacificus* sp. nov. and emended description of the genus *Thalassolituus*

**DOI:** 10.3389/fmicb.2022.1051202

**Published:** 2022-12-20

**Authors:** Chunming Dong, Lin Wei, Jianning Wang, Qiliang Lai, Zhaobin Huang, Zongze Shao

**Affiliations:** ^1^Key Laboratory of Marine Genetic Resources, Third Institute of Oceanography, Ministry of Natural Resources, Xiamen, China; ^2^State Key Laboratory Breeding Base of Marine Genetic Resources, Xiamen, China; ^3^Key Laboratory of Marine Genetic Resources of Fujian Province, Xiamen, China; ^4^College of Oceanology and Food Science, Quanzhou Normal University, Quanzhou, China

**Keywords:** *Oceanobacter*-related bacteria, phylogenomic, comparative genomic, taxonomic reassignment, *Thalassolituus hydrocarbonoclasticus*, *Thalassolituus pacificus*, hydrocarbon, marine carbon cycle

## Abstract

*Oceanobacter*-related bacteria (ORB) are a group of oligotrophic marine bacteria play an underappreciated role in carbon cycling. They have been frequently described as one of the dominant bacterial groups with a wide distribution in coastal and deep seawater of global oceans. To clarify their taxonomic affiliation in relation to alkane utilization, phylogenomic and comparative genomics analyses were performed based on currently available genomes from GenBank and four newly isolated strains, in addition to phenotypic and chemotaxonomic characteristics. Consistently, phylogenomic analysis robustly separated them into two groups, which are accordingly hydrocarbon-degrading (HD, *Thalassolituus* and *Oleibacter*) and non-HD (NHD, *Oceanobacter*). In addition, the two groups can also be readily distinguished by several polyphasic taxonomic characteristics. Furthermore, both AAI and POCP genomic indices within the HD group support the conclusion that the members of the genus *Oleibacter* should be transferred into the genus *Thalassolituus*. Moreover, HD and NHD bacteria differed significantly in terms of genome size, G + C content and genes involved in alkane utilization. All HD bacteria contain the key gene *alkB* encoding an alkane monooxygenase, which can be used as a marker gene to distinguish the members of closely related genera *Oceanobacter* and *Thalassolituus*. Pangenome analysis revealed that the larger accessory genome may endow *Thalassolituus* with the flexibility to cope with the dynamics of marine environments and thrive therein, although they possess smaller pan, core- and unique-genomes than *Oceanobacter.* Within the HD group, twelve species were clearly distinguished from each other by both dDDH and ANI genomic indices, including two novel species represented by the newly isolated strains alknpb1M-1*^T^* and 59MF3M-4*^T^*, for which the names *Thalassolituus hydrocarbonoclasticus* sp. nov. and *Thalassolituus pacificus* sp. nov. are proposed. Collectively, these findings build a phylogenetic framework for the ORB and contribute to understanding of their role in marine carbon cycling.

## Introduction

*Oceanobacter*-related bacteria (ORB), a group of bacteria within the order *Oceanospirillales* of *Gammaproteobacteria*, are closely related to *Oceanobacter kriegii* (Satomi et al., 2002), with more than 94% 16S rRNA gene sequence similarity. This group was first proposed by Teramoto and collaborators (Teramoto et al., 2009) and is now represented by three genera, i.e., *Oceanobacter* (Bowditch et al., 1984; Satomi et al., 2002; Huang et al., 2022), *Thalassolituus* (Yakimov et al., 2004), and *Oleibacter* (Teramoto et al., 2011). Currently, only a few strains of ORB have been isolated because of their resistance to cultivation, and these genera contain only one to three validly named species.

*Oceanobacter*-related bacteria (ORB) are an ubiquitous microbial clade in marine environments. 16S rRNA gene and metagenome-based surveys have shown that ORB, especially members of *Thalassolituus*, are widely distributed in marine environments *in situ*, such as seawater and sediments from various coastal seas and open oceans around the world (Yakimov et al., 2010), as well as iron–manganese concretions in the Baltic Sea (Yli-Hemminki et al., 2014), corals in the Andaman Sea (Badhai et al., 2016), and eggs and nauplii of the Pacific blue shrimp (Giraud et al., 2021). *Oleibacter* strains have been detected as the dominant members in the deep water of some marine basins where petroleum and gas activities occur (Miller et al., 2020), in biofilm communities on polyethylene pellets (Hansen et al., 2021) and polyvinyl chloride plates as pioneer colonizers (Pollet et al., 2018). In addition, previous studies have shown that *Oleibacter* is one of the dominant bacterial genera in the phycosphere of some marine microalgae (Chernikova et al., 2020) and in the bacterial community during phytoplankton blooms (Li et al., 2012). Moreover, several reports have shown that *Oceanobacter* can be isolated from coastal surface seawater (Baumann et al., 1972), mangrove sediment (Huang et al., 2022) and the phycosphere of some microalgae (Chernikova et al., 2020).

*Oceanobacter*-related bacteria (ORB) are clearly an ecologically important microbial clade in marine environments and are particularly involved in hydrocarbon metabolism. Notably, *Thalassolituus* and *Oleibacter* frequently occurred as predominant members in hydrocarbon-spiking microcosms of temperate seawater and sediment (Yakimov et al., 2005; McKew et al., 2007; Tremblay et al., 2017; Lofthus et al., 2018; Bôto et al., 2021), tropical seawater (Teramoto et al., 2009), oil plumes and seawater collected from the vicinity of the Deepwater Horizon blowout site (Hazen et al., 2010; Liu et al., 2017), Arctic and subarctic seawater (Dong et al., 2014; Suja et al., 2017; Shtratnikova et al., 2018) and sediment (Murphy et al., 2021), as well as in the *in situ* sediment after oil-spills (Lee et al., 2017; Thomas et al., 2020). Recently, they were identified as key hydrocarbon degraders not only in the ocean sunlight zone (Love et al., 2021) but also in the dark hadal zone (Liu et al., 2019; Jian et al., 2021). Interestingly, *Thalassolituus* and *Oleibacter* were also reported to benefit the most from trace metal additions (such as iron and zinc) during dark incubation experiments (Baltar et al., 2018). Collectively, these reports imply that the ubiquitous ORB, particularly *Thalassolituus* and *Oleibacter*, may play a substantial role in the element cycles of the ocean.

Based on phenotypic characteristics, particularly the ability to degrade hydrocarbons, ORB can be divided into two groups, the hydrocarbon-degrading group (*Thalassolituus* and *Oleibacter*, designated HD in this study) and the non-HD group (*Oceanobacter*, designated NHD). Members of the HD group grow slowly and form very small colonies on marine agar 2,216 plates (MA; BD Difco) and can oxidize alkanes in artificial seawater medium (Yakimov et al., 2004; Teramoto et al., 2011; Choi and Cho, 2013; Wei et al., 2022). In contrast, members of the NHD group grow quickly and form large colonies on MA plates but do not show the ability to utilize alkanes (Baumann et al., 1972; Teramoto et al., 2009; Huang et al., 2022). 16S rRNA gene-based phylogenetic analysis showed that ORB form an independent branch within the family *Oceanospirillaceae*. However, within this branch, HD *Oleibacter* always cluster with NHD *Oceanobacter* rather than HD *Thalsssolituus* (Teramoto et al., 2009; Choi and Cho, 2013; Huang et al., 2022). These inconsistencies between phenotypes and phylogenies indicate that an uncertain phylogenetic relationship may exist among the ORB. On the other hand, although more than sixty ORB strains and metagenome-assembled genomes (MAGs) are currently available in GenBank, almost 60% of them are not classified at the species level or are even incorrectly designated. As a result, misnamed taxa were also apparent in previous studies. For example, members of *Thalsssolituus* and *Oleibacter* were mistaken for *Oceanobacter* in a recent study, in which the abundance change of hydrocarbon-degrading genes was used to reflect whether an oil spill occurred (Bagi et al., 2022). In other words, the taxonomic affiliation of ORB species is not completely clarified according to previous studies. It is known that accurate taxonomic assignment is essential for making meaningful comparisons of bacterial physiology, metabolism, and genomic potential (Barco et al., 2020). Considering the important ecological role of ORB, phylogenetic and comparative genomics analyses based on the genomes are necessary to reevaluate and clarify their taxonomic affiliations to avoid confusion in subsequent ecological and evolutionary studies.

Accordingly, in this study, we established a whole-genome phylogeny for currently available high-quality genomes of ORB, coupled with genome-based relatedness indices, comparative genomics analysis, and consideration of phenotypic and chemotaxonomic differences to resolve taxonomic inconsistencies within these bacteria. In addition, we propose the reclassification of *Oleibacter marinus* into the genus *Thalassolituus*, describe two novel *Thalassolituus* species obtained in this study and amend the description of the genus *Thalassolituus*.

## Materials and methods

### Bacterial isolation and culture conditions

Four *Thalassolituus*-related strains originating from distinct marine habitats in three ocean regions were isolated in this study. Strains alknpb1M-1*^T^* and ST750PaO-4 were separately isolated from two consortia enriched with *Cyanobacteria*-derived alkanes (C_15_H_32_:C_17_H_36_ 9:1, v/v). These consortia were respectively set up with deep-sea sediment overlying water (−3,758 m, station MIES01, 18.49° N 116.27° E) and oxygen minimum zone (OMZ) seawater (−750 m, station SEAT, 18.00° N, 116.00° E) from the South China Sea during the China Ocean Mineral Resources R&D Association (COMRA) 45th cruise of the R/V Xiang-Yang-Hong-03 in July 2017 and the kk1904 cruise of the R/V Tan-Kah-Kee in June 2019. Strain 59MF3M-4*^T^* was isolated from crushed tissue of an unidentified deep-sea sponge (−1,790 m, station DY59-I-ROV11, 13.00° N 134.00° E) from the Western Pacific Ocean during the COMRA 59th cruise of the R/V Shen-Hai-Yi-Hao in August 2020. Strain 4BN06-13 was isolated from surface seawater enriched with crude oil from the Canada Basin (−3,566 m, station 4BN06, 81.46° N, 164.94° W) during the 4th Chinese National Arctic Research Expedition of the R/V Xulong icebreaker in August 2010. For the isolation of these strains, approximately 10^–4^, 10^–5^, and 10^–6^ dilutions of the enriched culture or the crushed sponge tissue were simultaneously spread onto ONR7a (supplemented with 0.1% alkanes, v/v) and MA (supplemented with 0.1% sodium acetate, w/v) media plates. The spread plates were then incubated at 15°C for 5–7 days in the dark until colonies emerged.

Since genome-based phylogeny and relatedness indices indicated that strains alknpb1M-1*^T^* and 59MF3M-4*^T^* represent two novel species, they were subjected to further polyphasic taxonomic analysis. For this purpose, three type strains, *Thalassolituus oleivorans* DSM 14913*^T^, Thalassolituus marinus* KCTC 23084*^T^* and *Thalassolituus alkanivorans* KCTC 82621*^T^*, were obtained from the German Collection of Microorganisms and Cell Cultures GmbH and Korean Collection for Type Cultures for comparative analysis in this study. Generally, the four isolates and the reference strains were routinely cultivated on modified MA plates or in Marine Broth 2,216 medium (MB, BD Difco™) supplemented with 1 g/L sodium acetate at 25°C.

### Genome sequencing and annotation

A total of five strains were subjected to genome sequencing analysis, including the four strains isolated in this study and the type strain *T. marinus* IMCC1826^T^. The complete genome sequence of strain alknpb1M-1^T^ was sequenced by using a paired-end (PE) 300-bp sequencing strategy on an Illumina Nova platform and a PacBio RS II platform with an insert length of 10 kb SMRTbell library (Hanyu Bio-Tech Co., Ltd., Shanghai, China). After filtering low-quality reads, clean PacBio subreads were assembled with the program HGAP (v.2.0) (Chin et al., 2013), and clean Illumina reads were used to correct the PacBio long reads and evaluate the complexity of the genome. The draft genome sequences of strains 59MF3M-4^T^, ST750PaO-4 and *T. marinus* IMCC1826^T^ were also obtained using the PE 300-bp sequencing strategy on the Illumina Nova platform and assembled with the velvet (v. 1.2.03) (Zerbino and Birney, 2008). The draft genome of strain 4BN06-13 was sequenced using a PE 500-bp strategy on an Illumina HiSeq2000 platform (Shanghai Majorbio Biopharm Technology Co., Ltd., Shanghai, China), and the high-quality reads were assembled with the SOAPdenovo (v.1.05) (Li et al., 2010). The quality of these genomes was estimated using CheckM (v1.0.12) (Parks et al., 2015). Gene prediction and annotation were performed using the Rapid Annotation using Subsystem Technology (RAST) pipeline (Aziz et al., 2008) and the Prokka (v1.13) (Seemann, 2014).

### Phylogeny analysis based on 16S rRNA gene and genomic sequences

The 16S rRNA gene sequences of newly isolated strains were retrieved from their genome sequences using the RNAmmer program (Lagesen et al., 2007). Similarities in 16S rRNA gene sequences between these taxa were determined using the EzBioCloud online server^1^ (Yoon et al., 2017). All available 16S rRNA gene sequences (> 1,315 bp) belonging to *Oceanobacter*-related strains (identity > 94.5%) were retrieved (until August 30, 2022) from GenBank using a BLASTN search (Altschul et al., 1997) and the strain alknpb1M-1^T^ full-length 16S rRNA gene sequence as the query sequence. Subsequently, thirty-two 16S rRNA gene sequences were obtained, and their closely related taxa were identified using the EzBioCloud online server. Additionally, twenty other 16S rRNA gene sequences of type strains of the type species for each genus within the family *Oceanospirillaceae* were also obtained from GenBank. The complete list of 16S rRNA gene sequences used in this study is provided in Supplementary Table 1. A phylogenetic tree based on the 16S rRNA gene sequences was generated using the maximum likelihood method with the program MEGA (v.10.0.5) (Kumar et al., 2018). Evolutionary distances were calculated using Kimura’s two-parameter model (Kimura, 1980). Bootstrap analysis based on 1,000 replicates was used to estimate the node robustness. The sequence of *Litoricola lipolytica* IMCC1097^T^ was used as the outgroup.

For phylogenomic analysis, we collected all currently available genomes belonging to the ORB and the type strains of type species for each genus within the family *Oceanospirillaceae* (until August 30, 2022) from GenBank. In particular, MAGs related to the ORB were also included considering the scarcity of the cultivated ORB strains. Including the 5 genomes sequenced in this study, a total of 77 genomes were obtained (Supplementary Table 2). The qualities of these genomes were also estimated using CheckM. Finally, 54 genomes met the quality-control criteria (completeness > 90% and contamination < 5%) and were chosen for the subsequent phylogenomic analysis and genome-based relatedness index calculation. Genome-based phylogenetic analysis was performed using the Genome Taxonomy Database Toolkit (GTDB-Tk, v. 1.5.0) (Chaumeil et al., 2019) along with the Genome Taxonomy Database (v. R202) (Parks et al., 2018, 2020) with default parameters. *L. lipolytica* IMCC1097^T^ was selected as the outgroup. The phylogenomic tree and support values were visualized using MEGA (v.10.0.5). Moreover, the taxonomies of several MAGs obtained from GenBank may be misnamed owing to incomplete classification within the family *Oceanospirillaceae* at present; thus, their taxonomy inferred by the GTDB-Tk is provided in Supplementary Table 2. Additionally, a phylogenetic splits network of the ORB was reconstructed by SplitsTree (v. 4.18.3) using the neighbor-net algorithm (Huson and Bryant, 2006). This network was based on the concatenated sequences of five housekeeping genes, including *gyrB* (gyrase B subunit), *polA* (DNA polymerase I), *icd* (isocitrate dehydrogenase), *pryH* (uridylate kinase), and *mdh* (malate dehydrogenase).

### Genome-based relatedness indices for genus and species delineation

To clarify the affiliation of ORB at the genus level, the average amino acid identity (AAI) (Konstantinidis and Tiedje, 2005) and percentage of conserved proteins (POCP) (Qin et al., 2014) were used for amino acid level comparisons for every pairwise combination of genomes. AAI values were determined using CompareM v0.0.23 (Parks, 2016), whereas POCP values were calculated using a Python script (Liang, 2019) with the formula [(C1 + C2)/(T1 + T2)] × 100%, where C1 and C2 represent the number of conserved proteins in the two genomes, and T1 and T2 represent the total number of proteins in the two genomes, respectively. Two genomes belonging to the same genus typically have an AAI value of at least 65–72% (Konstantinidis and Tiedje, 2007), corresponding to at least 50% POCP (Qin et al., 2014). To clarify the affiliations of ORB at the species level, digital DNA-DNA hybridization (dDDH) (Meier-Kolthoff et al., 2013) and average nucleotide identity (ANI) (Goris et al., 2007) were used for nucleotide-level comparisons for every pairwise combination of genomes. The dDDH values were calculated using the TYGS online service^2^ (Meier-Kolthoff and Göker, 2019). ANI values were calculated using fastANI v1.3 (Jain et al., 2018) under default parameters. Two genomes belonging to the same species are expected to have a dDDH value of at least 70% (Meier-Kolthoff et al., 2013), corresponding to at least 95–96% ANI (Goris et al., 2007). Visualization of the numerical matrices for AAI, POCP, dDDH, and ANI values was performed using TBtools v1.0987663 (Chen et al., 2020).

### Pangenomics analyses

Pangenome analysis for the ORB genomes was performed by the BPGA pipeline (Chaudhari et al., 2016) with default parameters to obtain the inter species variation and pangenome and core genome profiles. The GenBank format annotation files generated by the Prokka program were used as input files. Orthologous clusters were assigned by clustering all protein sequences in the analyzed genomes using USEARCH based on their sequence similarity (> 50% cut off). Through pangenome analysis, we compiled a set of core genes shared among all strains, a set of distributed genes shared with more than two but not all strains, and unique genes only found in a single strain. In addition, the genomes affiliated with the HD and NHD groups were also utilized to obtain pangenome profiles. Clusters of orthologous gene (COG) distributions of the core, accessory and unique gene families were based on representative sequence annotation results using EggNOG-mapper (v2.1.9) (Cantalapiedra et al., 2021), and the significance difference in COG distribution between the HD and NHD groups was determined with STAMP (v2.1.3) (Parks et al., 2014) using Fisher’s exact test (*p* < 0.05).

### Identification and phylogeny analysis of genes involved in alkane metabolism

The proteins involved in alkane degradation, including alkane monooxygenase (AlkB), Baeyer-Villiger monooxygenase (BVMO), ferredoxin and ferredoxin reductase, alcohol dehydrogenase, aldehyde dehydrogenase and esterase, were identified using a local BLASTP search program with the amino acid sequences listed in Supplementary Table 3 as queries. These queried protein sequences were extracted from strain *T. oleivorans* MIL-1^T^, and their functions have all been verified by proteomics analysis combined with the detection of the hydrocarbon-derived metabolites catalyzed by these proteins (Gregson et al., 2018). For the BLASTP analysis, we used an amino acid similarity cutoff of 40%, alignment coverage > 80%, and an *e*-value cutoff of 1e^–5^. The presence/absence of these protein-coding genes in the genomes of ORB strains was further checked based on functional annotation by RAST. Additionally, given the importance of the proteins AlkB and BVMO in the process of alkane initial degradation, they were selected for phylogenetic analysis. The phylogenetic tree reconstruction was inferred using the neighbor-joining method (Saitou and Nei, 1987) with MEGA (v.10.0.5) based on their amino acid sequences. Genetic distances for these analyses were calculated using the Poisson model (Zuckerkandl and Pauling, 1965). Bootstrap analysis was performed with 1,000 resamples.

### Physiology and chemotaxonomic characteristics

The novel strains alknpb1M-1^T^ and 59MF3M-4^T^ were subjected to physiological and chemotaxonomic analyses. Cell and colony morphology, Gram staining, motility, and hydrolysis of Tween 80 and starch were determined according to the methods of Dong and Cai (2001). Oxidase activity was evaluated using oxidase detection strips supplied by Oxoid Ltd., (Oxoid Limited, Basingstoke, UK), and catalase activity was determined by applying 1% (v/v) hydrogen peroxide. Anaerobic growth and growth temperature, NaCl tolerance, and pH ranges were tested according to previously described methods (Dong et al., 2021). Other biochemical tests for these strains and three reference strains were carried out using API 20NE, 20E, and ZYM strips (bioMérieux, France) according to the manufacturer’s instructions, and the NaCl concentration was adjusted to 3.0%. The ability of the two strains to use hydrocarbons was examined in ONR7a medium (Dyksterhouse et al., 1995) supplemented with 0.3% (v/v) of various chain-length alkanes as the sole carbon and energy source at 25°C for up to 4 weeks in the dark. Growth status was monitored using a spectrophotometer at 600 nm. In this study, the following hydrocarbons were tested: C_8_H_18_, C_15_H_32_, C_16_H_34_, C_24_H_50_, C_32_H_66_ and a mixture (C_15_H_32_:C_17_H_36_ 9:1, v/v) to simulate the photosynthetic hydrocarbons produced by *Cyanobacteria* in the open ocean.

The fatty acid profiles of the two strains and the three type strains were determined in parallel using cells from the third quadrants on modified MA medium at 25°C for 72 h. Cellular fatty acids were saponified, methylated, and extracted according to the standard MIDI (Sherlock Microbial Identification System, version 6.0B) protocol. The fatty acids were then analyzed by gas chromatography (Agilent Technologies 6850) and identified using the TSBA6.0 database of the Microbial Identification System (Sasser, 1990). Quinone and polar lipids were analyzed according to previously described methods (Collins, 1985; Kates, 1986). Additionally, the abovementioned characteristics of the type strains of the genera *Oceanobacter* (Bowditch et al., 1984; Satomi et al., 2002; Huang et al., 2022) and *Oleibacter* (Teramoto et al., 2011) were collected from their original reports.

## Results and discussion

### Phylogeny revisit based on 16S rRNA gene sequences

**FIGURE 1:**
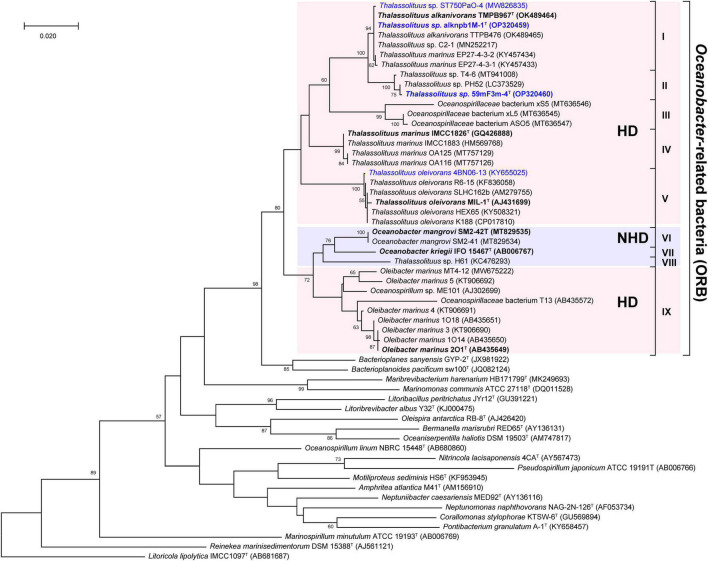
Maximum-likelihood tree showing the phylogenetic relationships of *Oceanobacter*-related bacteria along with other type strains of type species within the family *Oceanospirillaceae* based on 16S rRNA gene sequences. Bootstrap values (expressed as percentages of 1,000 replicates) are shown at branch points. Strains isolated in this study and the type strains of ORB are highlighted in blue and black bold, respectively. Bar, 0.02 nucleotide substitution rate (Knuc) units. *Litoricola lipolytica* IMCC1097^T^ (AB681687) was used as the outgroup.

To more comprehensively determine the taxonomic status of ORB, phylogenetic analyses were performed using 16S rRNA gene sequences of all 36 type and representative strains of this group, together with another 20 type strains within the family *Oceanospirillaceae* (Supplementary Table 1). First, most of the pairwise 16S rRNA gene sequence similarity among the ORB are more than 94% (Supplementary Figure 1), which is very close to the 94.5% borderline of genus delineation (Yarza et al., 2014), indicating that these ORB seem to be assigned to the same genus. Furthermore, the maximum-likelihood phylogenetic tree also showed that ORB formed an independent branch with a high bootstrap value. As shown in Figure 1, this independent branch can be divided into nine clades (I–IX), which may represent at least nine species within the ORB. Clades I-V represent the two novel species proposed in this study (I and II), another novel candidate *Thalassolituus* species (III), the previously established species of *T. marinus* (IV), and *T. oleivorans* (V). Clades VI-VIII contained isolates related to the species *Oceanobacter mangrovi* (VI) and *Oceanobacter kriegii* (VII) and a novel candidate *Oceanobacter* species (VIII), respectively. Clade IX contained the strains related to the previously established genus *Oleibacter*. Compared to their close relatives, all these nine clades seemed more likely to belong to the same genus and represent different species, because this ORB branch exhibited phylogenetic depth similar to that of the neighboring genera *Bacterioplanes* and *Bacterioplanoides* and may also be considered a monophyletic taxon (Figure 1). These results are consistent with previous observations based on several type strains of genera within the ORB (Teramoto et al., 2009, 2011; Choi and Cho, 2013; Huang et al., 2022).

It is known that a monophyletic taxon is composed of all descendants of a common ancestor, and the monophyletic characteristics of members in a phylogenetic tree are the main criteria defining a taxon (Rosselló-Mora and Amann, 2001). However, although members of *Thalassolituus, Oleibacter*, and *Oceanobacter* were clustered together, they have several distinct phenotypic characteristics described in the Introduction section and are thought to be affiliated with two different bacterial groups. Overall, these inconsistencies between the 16S rRNA gene-based phylogeny and phenotype suggested an uncertain phylogenetic relationship among ORB.

### Phylogenomic analysis

**FIGURE 2:**
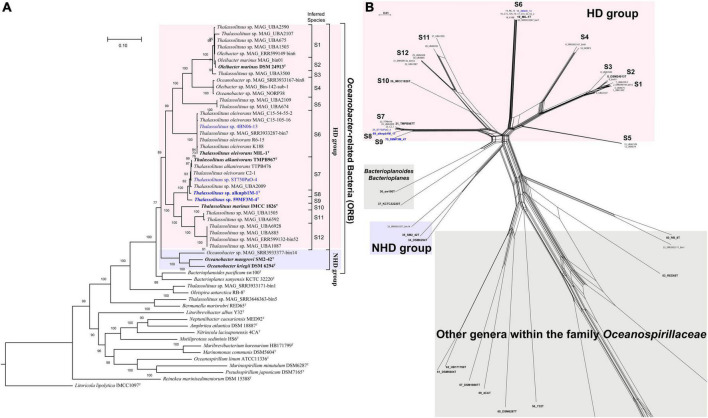
Phylogenetic relationships of the *Oceanobacter*-related type or representative strains and metagenome-assembled genomes (MAGs) along with the type strains within the family *Oceanospirillaceae*. **(A)** The maximum-likelihood phylogenomic tree was constructed using the concatenation of 120 ubiquitous bacterial single-copy proteins identified by GTDB-Tk (v. 1.5.0). The inferred taxonomies of all genomes are shown in Supplementary Table 2. Bootstrap values converted to a percentile scale were based on 1,000 replicates. Only bootstraps exceeding 50% are shown. Genomes retrieved from the strains isolated in this study and the type strains of ORB are highlighted in blue and black bold, respectively. Genomes retrieved from the metagenome-assembled genomes are marked by “MAG” in their names. The type strain *Litoricola lipolytica* IMCC1097^T^ was used as the outgroup. **(B)** Phylogenetic network of *Oceanobacter*-related bacteria reconstructed by SplitsTree (v. 4.18.3) using the neighbor-net algorithm. The network was based on the concatenated sequences of five housekeeping genes, including *gyrB* (gyrase B subunit), *polA* (DNA polymerase I), *icd* (isocitrate dehydrogenase), *pryH* (uridylate kinase), and *mdh* (malate dehydrogenase).

Unlike the 16S rRNA gene-based tree, genome-based phylogenetic analysis provided a clear topology structure for the ORB. Using GTDB-Tk, a whole-genome-based phylogenetic tree was constructed using all currently available high-quality genomes from the type or representative strains and MAGs within the family *Oceanospirillaceae*, together with the five genomes sequenced in this study. The phylogenomic tree revealed robust bootstrap support for most branches (Figure 2A). Notably, members of *Thalassolituus* and *Oleibacter* were clustered together, formed a monophyletic branch and separated from *Oceanobacter* in the family *Oceanospirillaceae* with a high bootstrap support value, indicating that they should be assigned to the same genus. The phylogenetic splits network result (Figure 2B) was also consistent with the phylogenomic analysis (Figure 2A). Therefore, these results further highlighted apparent taxonomic inconsistencies with those observed in the 16S rRNA gene-based tree and enabled the confident establishment of relationships within ORB. Subsequently, the inferred phylogenomic tree, together with the following genome-based relatedness indices, was used to assess the monophyletic status of members supposedly belonging to the same taxon.

As shown in Figure 2A, the species within the ORB were assigned to two groups, HD and NHD. Group HD was the largest and contained all genomes retrieved from the genera *Thalassolituus* and *Oleibacter*. This group could be further divided into twelve clades (S1–S12). Among these clades, clade S6 was the largest and consisted of four isolated strains and three MAGs, which were all retrieved from hydrocarbon-enriched consortia (Supplementary Table 2). The four strains MIL-1^T^, K188, R6-15, and 4BN06-13 were separately isolated from seawater obtained from the Italian Sicily coastal harbor (Yakimov et al., 2004), Barents Sea (Shtratnikova et al., 2018), Chukchi Sea (Dong et al., 2014), and Canada Basin. The MAG SRR3933287_bin7 originated from the oil-enriched coastal seawater of Canada. The other two MAGs were obtained from *Cyanobacteria*-derived alkane-enriched northern Atlantic oceanic mesopelagic seawater (Love et al., 2021). Both clades S7 and S1 contained five members. In clade S7, the type strain *T. alkanivorans* TMPB967^T^ together with two other strains (TTPB476 and C2-1) were isolated from deep seawater or sediment of the Mariana Trench. Notably, previous reports indicated that large amounts of alkanes were periodically released from sediments to seawater in this trench (Li et al., 2018; Liu et al., 2019). Therefore, these trench-originated bacteria likely benefit from periodic alkane pulses. In addition, strain ST750PaO-4 and MAG UBA2009 were obtained from the *Cyanobacteria*-derived alkane-enriched OMZ seawater of the South China Sea and a hydrothermal plume in the Mid Cayman Rise of the Atlantic Ocean, respectively. Clade S1 was composed entirely of uncultivated bacteria, which were all retrieved from the surface to mesopelagic seawater in the South and North Atlantic Gyre by the Tara Ocean project (Sunagawa et al., 2015), except MAG ERR599149-bin6. Clades S12, S11, and S5 included four, two and two uncultivated bacteria, respectively. Except MAG ERR599132_bin52, all other members of these clades were also retrieved from the surface to mesopelagic seawater from various ocean regions by the Tara Ocean project. Clade S4 included three MAGs, which were separately retrieved from the coastal seawater of Germany or Canada and the cold oxic subseafloor aquifer of the Atlantic Ocean (Tully et al., 2018). Clade S2 included the type strain *Oleibacter marinus* DSM 24913^T^ (Teramoto et al., 2011) and MAG bin01 from Mariana Trench hadal water (Liu et al., 2019). Clade S3 contained MAG UBA3500, which was retrieved from the deep chlorophyll maximum layer seawater of the Red Sea (Sunagawa et al., 2015). Clades S8–S10 were all monophyletic branches and separately contained one strain. In these clades, strains alknpb1M-1^T^ and 59MF3M-4^T^ represented two candidate novel species, while strain IMCC1826^T^ is the type strain of *T. marinus* (Choi and Cho, 2013). Overall, these abovementioned environmental sources of the HD bacteria further indicated that the members of *Thalassolituus* and *Oleibacter* are likely involved in hydrocarbon metabolism not only in laboratory-enriched consortia but also in *in situ* marine environments. In contrast, group NHD only contained two type strains within the genus *Oceanobacter* and a MAG, which all originated from coastal seawater or sediment (Supplementary Table 2).

### Use of AAI and POCP for genus delineation

**FIGURE 3:**
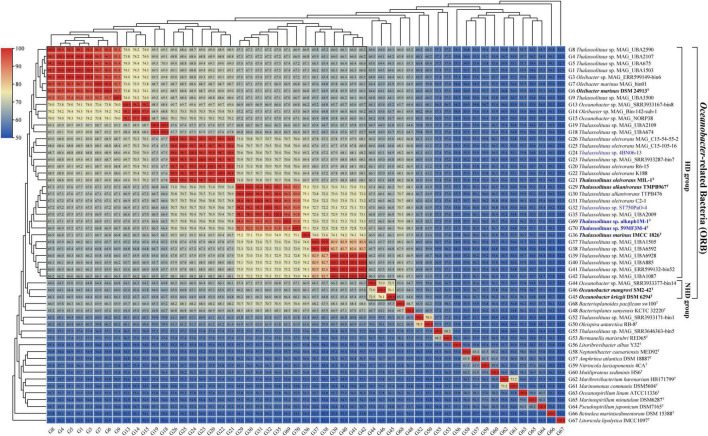
Amino acid identity (AAI) value matrices from pairwise genome comparisons. The heatmap shows AAI values (in each color block) between genomes, along with the phylogenomics tree (Figure 2A) cladogram to show relationships. The boxed regions indicate inferred genera within the *Oceanobacter*-related bacteria (ORB) based on AAI comparisons, as well as monophyly in the genome-based phylogenetic tree (Figure 2A). Similarly, inferred genera were also observed from the percentage of conserved proteins (POCP) value matrices (Supplementary Figure 2). Genomes retrieved from the strains isolated in this study and the type strains of ORB are highlighted in blue and black bold, respectively. Genomes retrieved from the metagenome-assembled genomes are marked by “metagenome-assembled genomes (MAG)” in their names.

Amino acid identity (AAI) thresholds between 65 and 72% (Konstantinidis and Tiedje, 2007) or 60–80% (Luo et al., 2014) have been proposed to determine whether a species belongs to two different genera. Clearly, these are large ranges in use for genus delineation. Therefore, specific AAI thresholds have been proposed for several families for genus delineation based on calculated AAI values. For example, in the families *Methylothermaceae* (Skennerton et al., 2015), *Methylococcaceae* (Orata et al., 2018) and *Rhodobacteraceae* (Wirth and Whitman, 2018), 70, 71, and 80% AAI can be used as the lower genus limits, respectively. In this study, the AAI values within the HD group (range 64.5–100%, mean 73.2%) were higher than those between the members of the HD and NHD groups (range 63.2–67.4%, mean 65.2%) (Figure 3). When only considering the cultured strains, they shared 66.8–100% of AAI within the HD group and shared 64.1–66.4% of AAI between the two groups (Supplementary Table 4). Therefore, the minimum difference from 66.8% AAI observed within the cultured HD members could be used as a candidate threshold to distinguish different genera in the ORB. This threshold falls within the previously proposed range (65–72%) described by Konstantinidis and Tiedje (2007). Notably, there were several AAI values (64.5–66.8%) slightly lower than 66.8% among some MAGs and strains in the HD group (Figure 3). Considering that they were all draft genomes, these AAI values may have been underestimated during the calculation.

Additionally, a POCP threshold of at least 50% was proposed to determine whether the two species belong to the same genus (Qin et al., 2014). However, several recent studies have suggested that a 50% POCP threshold for genus delineation is overly stringent and should be reevaluated, such that higher values (typically in the range 58–66%) may more appropriate (Aliyu et al., 2016; Orata et al., 2018; Wirth and Whitman, 2018; Gupta, 2019; de la Haba et al., 2021; Xu et al., 2021; Sangal et al., 2022). In this study, the POCP values within the HD group (range 55.2–99.4%, mean 69.2%) were all above 50% and higher than those between the members of the HD and NHD groups (range 46.3–60.4%, mean 54.3%) (Supplementary Figure 2). These values support the conclusions that the HD members (*Thalassolituus* and *Oleibacter*) could be assigned to the same genus and they are separated from other ORB. Furthermore, when only considering the cultured strains, they shared 60.4–98.6% of POCP within the HD group and shared 48.9–55.8% of POCP between the two groups (Supplementary Table 4). Similar to the AAI, we propose that the minimum POCP value (60.4%) observed within the cultured HD members as the threshold can be used for genus delineation in the ORB. This threshold value is in line with the abovementioned range (58–66%).

Collectively, both the AAI and POCP indices supported that the members of *Thalassolituus* and *Oleibacter* (HD group) belong to the same genus. These results are consistent with the genome-based phylogenetic analyses (Figure 2). Thus, we propose the transfer of *Oleibacter* species into the genus *Thalassolituus*, as the latter was the first established genus within the HD group (Yakimov et al., 2004).

### Use of dDDH and ANI for species delineation

**FIGURE 4:**
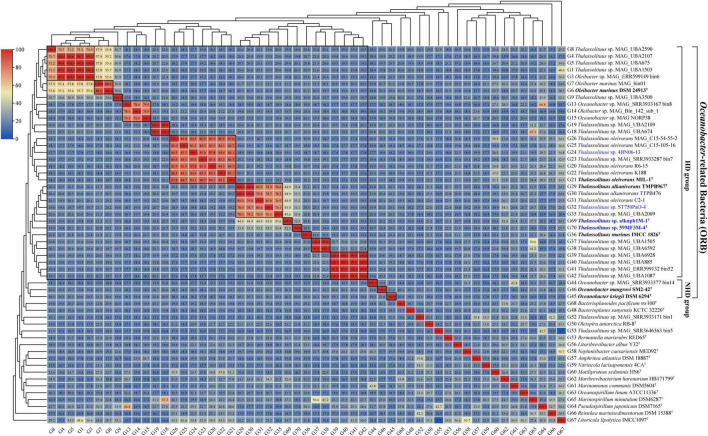
digital DNA-DNA hybridization (dDDH) value matrices from pairwise genome comparisons. The heatmap shows dDDH values (in each color block) between genomes, along with the phylogenomics tree (Figure 2A) cladogram to show relationships. The largest boxed region indicates the revised genus *Thalassolituus*. Twelve small boxed regions indicate the inferred species clusters within the revised genus *Thalassolituus* based on dDDH comparisons, as well as monophyly in the genome-based phylogenetic tree (Figure 2A). Identically inferred species clusters were also observed from the average nucleotide identity (ANI) value matrices (Supplementary Figure 3). Genomes retrieved from the strains isolated in this study and the type strains of *Oceanobacter*-related bacteria (ORB) are highlighted in blue and black bold, respectively. Genomes retrieved from the metagenome-assembled genomes are marked by “metagenome-assembled genomes (MAG)” in their names.

Once the members of the genus *Oleibacter* were reclassified into the genus *Thalassolituus*, misclassifications at the species level within this newly expanded genus need to be addressed. According to generally accepted criteria, in this study, the genomes were considered to belong to the same species if they had a dDDH of at least 70%, an ANI of more than 95%, and clustered in a monophyletic clade in the genome-based phylogeny (Rosselló-Mora and Amann, 2001). Subsequently, the dDDH values were calculated for all genomes from the HD group members. The 34 genomes within the genus can be divided into 12 distinct species clusters (Figure 4, 12 small boxed regions), which exactly corresponded to the 12 monophyletic clades (clades S1-S12) observed in the phylogenomic tree (Figure 2A). Furthermore, these observations were fully supported by the data obtained using ANI analysis (Supplementary Figure 3). In particular, six clades shown in Figure 2A all contained at least one cultivable strain, with S2 (*O. marinus*), S6 (*T. oleivorans*), S7 (*T. alkanivorans*), and S10 (*T. marinus*) representing previously described species, and two novel species within S8 and S9 represented by strains isolated in the present study.

Within the four strains isolated in this study, strains alknpb1M-1^T^ and 59MF3M-4^T^ formed monophyletic branches separated from the other HD members in the phylogenomic tree (Figure 2A, S8 and S9 respectively). The dDDH values of the two strains with *T. oleivorans* MIL-1^T^, *T. marinus* IMCC1826^T^ and *T. alkanivorans* TMPB967^T^ were 19.8–44.9% and 19.9–33.4% (Figure 4), respectively. The corresponding ANI values were 78.1–93.0% and 78.4–89.1% (Supplementary Figure 3). Meanwhile, the dDDH and ANI values between strains alknpb1M-1^T^ and 59MF3M-4^T^ were 32.3 and 88.8%, respectively. Therefore, these results indicated that these two strains represent two novel species of the genus *Thalassolituus.* In contrast, both the dDDH and ANI values of the other two strains isolated here, ST750PaO-4 and 4BN06-13, with the respective type strains demonstrated that they belong to the species *T. alkanivorans* and *T. oleivorans*, respectively.

### Genomic features and pangenome of the ORB

The genome sizes of the four analyzed strains alknpb1M-1^T^, 59MF3M-4^T^, ST750PaO-4, and 4BN06-13 were 4.07, 4.27, 4.28, and 3.69 Mb, which are similar sizes to those of three type strains of the genus *Thalassolituus* (3.91–4.37 Mb), and contained 3,710, 3,982, 3,988, and 3,512 annotated protein-coding genes, respectively. The genomic G + C content of the four strains was 46.5–53.4%, which was in line with that of the related type strains (46.4–53.1%). The detailed genomic characteristics of the four analyzed strains and other ORB strains and MAGs are listed in Supplementary Table 2. For all ORB members, groups HD and NHD showed significant (*P* < 0.05) differences not only in genome size but in G + C content (Supplementary Figure 4). More specifically, compared to the NHD group, HD members possess smaller genomes (3.3–4.4 Mb) and lower G + C content (46.5–55.1%). These results highlighted the genomic differences between HD and NHD members.

**FIGURE 5:**
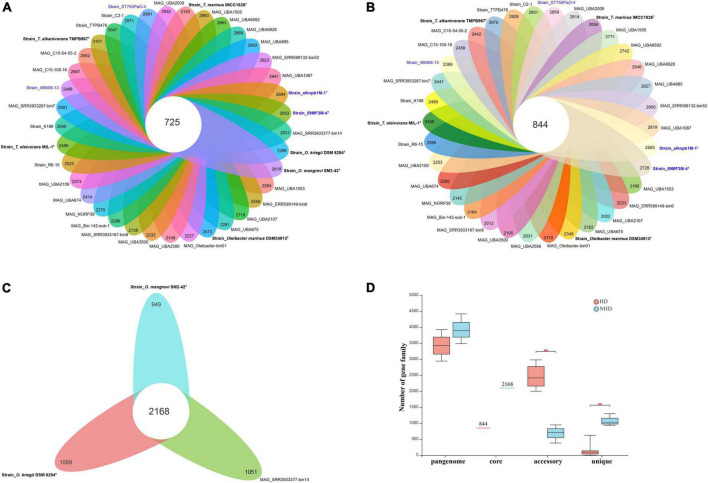
The pangenomes of *Oceanobacter*-related bacteria (ORB) strains. **(A)** Flower plots showing the core gene number (in the center) and the accessory gene numbers (in the petals) in the 37 strains. **(B)** Flower plots showing the core gene number (in the center) and accessory gene number (in the petals) in the hydrocarbon-degrading (HD) group. **(C)** Flower plots showing the core gene number (in the center) and accessory gene number (in the petals) in the non-hydrocarbon-degrading NHD group. **(D)** Variations in the numbers of pangenome, core, accessory, and unique genes between the HD and NHD groups. Significant levels in variations were determined using the Wilcoxon test (**P < 0.01) except for the core gene.

Pangenomic analysis was performed to investigate the genotypic features of all ORB, HD, and NHD members. The pangenome of the 37 ORB genomes contained 18,127 genes, including 725 core genes (Table 1, Figure 5A). The core genes accounted for only 3.9% of the pangenome and ranged from 16.4 to 24.3% in each genome, indicating that the ORB strains share a low percentage of common functional proteins. For accessory genes, a total of 11,010 genes were found, which accounted for 59.5% of the pangenome and ranged from 57.9 to 80.8% in each genome. Accessory genes usually offer bacterial species diversity, environmental adaptation and other characteristics (Innamorati et al., 2020). Such a high proportion of accessory genes may partly explain why they are widely distributed in marine environments. Strain-unique genes are those that are present in only one strain and are thought to be derived *via* horizontal gene transfer (Bentley, 2009); these genes accounted for 36.6% of the pangenome genes among all ORB bacteria, and the number of unique genes varied from 1 to 1,079 among different strains.

**TABLE 1:** Pangenome size of *Oceanobacter*-related bacteria and its different groups.

	All ORB	HD group	NHD group
Pangenome genes	18,127	14,582	6,439
Core genes	725 (3.9%)	844 (5.8%)	2,168 (33.7%)
Accessory genes	11,010 (59.5%)	9,542 (65.4%)	1,023 (15.9%)
Unique genes	6,764 (36.6%)	4,196 (28.6%)	3,248 (50.4%)

To understand the relationships between pangenome size, core gene number, and strain prevalence of the ORB bacteria, we plotted the fitted curves of the pangenome profile of the 37 genomes. As shown in Supplementary Figure 5A, the number of conserved genes detected upon the sequential addition of each new genome was inferred by fitting a decaying function (pink curve), suggesting that the average number of core genes approached a relatively constant number. In contrast, the blue curve increased with the addition of a new genome and was far from saturation, indicating that the genetic repertoire of the species was still growing. Thus, the pangenome of the ORB bacteria is open. Similarly, some environmental representative bacteria, such as *Streptococcus* (Tettelin et al., 2005), *Shewanella* (Konstantinidis et al., 2009), and *Francisella* (Kumar et al., 2020), have open pangenomes.

To detect the genotypic differentiation between HD and NHD members, their pangenome components were compared (Figures 5B, C). We found that the members of the two groups contained distinct numbers of pangenome and core genes and exhibited significant differences in the numbers of accessory (*p* < 0.01) and unique (*p* < 0.01) genes (Figure 5D). More specifically, compared to NHD, HD members exhibited smaller pangenome sizes, more accessory genes and fewer unique genes. The fitted curves of the pangenome profile of the HD and NHD genomes were also far from saturation (Supplementary Figures 5B, C, blue curve). Thus, similar to all ORB strains, the pangenomes of HD and NHD bacteria are both open, indicating that these strains will continue to gain genes and keep evolving.

To survey the functions of the genes that constitute the pangenome, COG functional classification was performed. Generally, HD and NHD bacteria showed high similarity in terms of COG category proportions in the core genome except for the genes categorized as unclassified by EggNOG-mapper (-) (Supplementary Figure 6A), indicating that the core genes of the two groups are similar. In the accessory genome, unclassified by EggNOG-mapper (-), replication, recombination and repair (L), and cell cycle control, cell division (D) genes were overrepresented (*p* < 0.05) in HD bacteria. However, NHD bacteria were significantly (*p* < 0.05) enriched in amino acid transport and metabolism (E), energy production and conversion (C), inorganic ion transport and metabolism (P), translation, ribosomal structure and biogenesis (J), and carbohydrate transport and metabolism (G) among the COG categories (Supplementary Figure 6B). These results suggested that NHD bacteria are more effective than HD bacteria in terms of exogenous nutrient acquisition (such as amino acids, carbohydrate and ions) and energy production, which is consistent with their fast growth rate and large colonies on medium plates. Moreover, NHD bacteria contained more unique genes within the following COG categories: carbohydrate transport and metabolism (G), inorganic ion transport and metabolism (P), signal transduction mechanisms (T), energy production and conversion (C), transcription (K), amino acid transport and metabolism (E), and function unknown (S). These unique genes may further endow NHD bacteria with the ability to obtain nutrients and energy from the surroundings. In contrast, HD bacteria were only significantly (*p* < 0.05) enriched in some unclassified genes (-), replication, recombination and repair (L), and cell wall/membrane/envelope biogenesis (M) (Supplementary Figure 6C). Notably, the unclassified (-) and replication, recombination and repair (L) genes were significantly (*p* < 0.05) enriched in both the accessory and unique genomes of the HD group, indicating that HD bacteria possess more currently unknown novel genes and possibly undergo more environmental stresses than NHD bacteria.

### Alkane metabolism of ORB

Terminal or subterminal oxidation is the initiating step of alkane degradation, which converts alkanes to primary or secondary alcohols, respectively. These alcohols are further oxidized to the corresponding aldehyde or ketone, and finally converted into fatty acids. Fatty acids are finally oxidized to CO_2_ *via* beta-oxidation (Singh et al., 2012). In this study, genomic analysis revealed that both the four analyzed strains and all other members in the HD group contain diverse alkane degrading-related genes, which encode AlkB, BVMO, ferredoxin and ferredoxin reductase, dehydrogenases, and esterase. In contrast, all of the NHD members are missing the *alkB* genes, which encode the key protein responsible for the initiation of alkane degradation.

**FIGURE 6:**
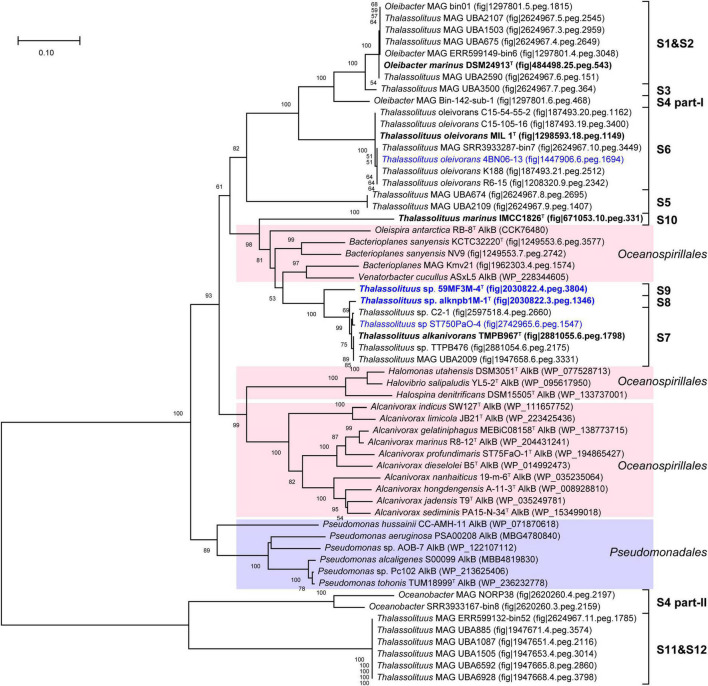
Neighbor-joining phylogenetic tree of alkane monooxygenase AlkB1 protein sequences derived from hydrocarbon-degrading (HD) group strains of the *Oceanobacter*-related bacteria (ORB) and their closest reference sequences. Bootstrap values (> 50%) based on 1,000 resamplings are given at the nodes. The labels in blue and black bold font indicate the sequences obtained from the strains isolated in this study and the type strains of the ORB, respectively. Reference sequences in different shades of color indicate that they originated from different bacterial orders: red, *Oceanospirillales*; purple, *Pseudomonadales*.

**FIGURE 7:**
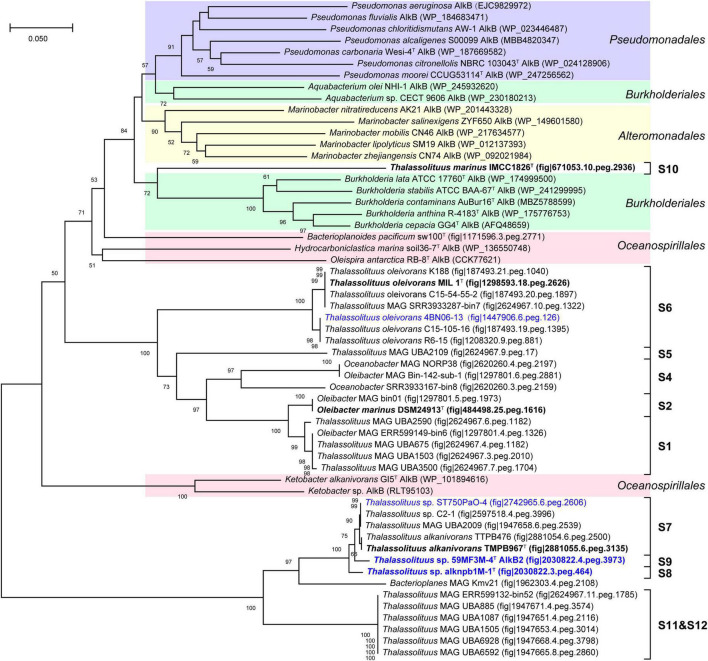
Neighbor-joining phylogenetic tree of alkane monooxygenase AlkB2 protein sequences derived from hydrocarbon-degrading (HD) group strains of the *Oceanobacter*-related bacteria (ORB) and their closest reference sequences. Bootstrap values (> 50%) based on 1,000 resamplings are given at the nodes. The labels in blue and black bold font indicate the sequences obtained from the strains isolated in this study and the type strains of the ORB, respectively. Reference sequences in different shades of color indicate that they originated from different bacterial orders: red, *Oceanospirillales*; purple, *Pseudomonadales*; yellow, *Alteromonadales*; green, *Burkholderiales*.

Alkane monooxygenase (AlkB) catalyzes alkanes to alkanols (van Beilen et al., 1994). In this study, two AlkBs (AlkB1 and AlkB2) are present in all HD members but absent in all NHD (Supplementary Table 5), suggesting that AlkB is essential to these HD bacteria for alkane degradation. Furthermore, AlkB1 and AlkB2 from HD members separately have 30.4–100% and 55.7–100% amino acid sequence identities to their counterparts CCU71603 (GenBank accession number) and CCU73056 in strain *T. oleivorans* MIL-1^T^ (Gregson et al., 2018), indicating that AlkB2 is more conserved than AlkB1 in the HD group. Phylogenetic analysis showed that both the AlkB1 and AlkB2 trees (Figures 6, 7) had similar topologies to the genome-based phylogenetic tree (Figure 2A), implying that they were probably coevolving with the genomes. In the AlkB1 tree, the closest AlkB homologs of HD members were mainly retrieved from the species in the orders *Oceanospirillales* and *Pseudomonadales*. However, in the AlkB2 tree, the closest homologs were retrieved from more diverse species, including those from the orders *Pseudomonadales, Alteromonadales*, and *Oceanospirillales* within the *Gammaproteobacteria* and the order *Burkholderiales* within the *Betaproteobacteria*. These different taxonomic origins of the closest homologs indicated that AlkB1 and AlkB2 may have different evolutionary origins and routes.

**FIGURE 8:**
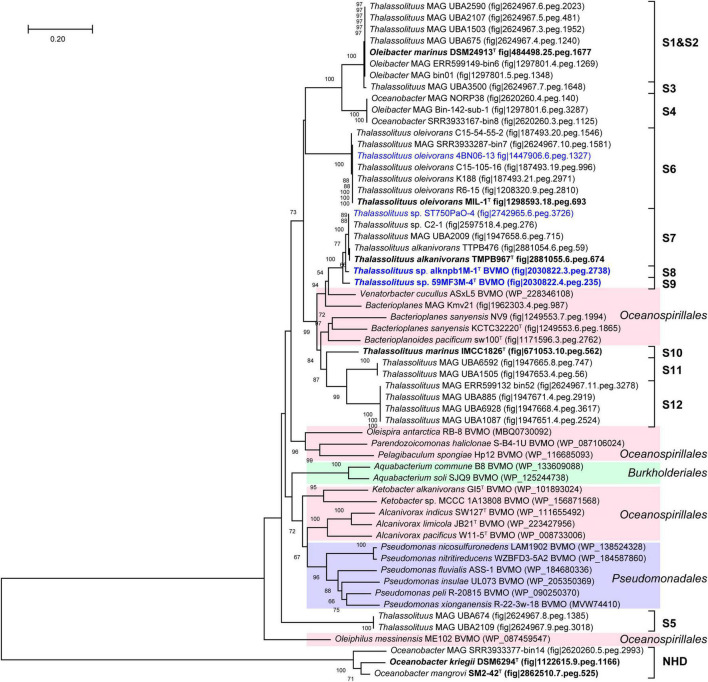
Neighbor-joining phylogenetic tree of Baeyer-Villiger monooxygenase Baeyer-Villiger monooxygenase (BVMO) protein sequences derived from *Oceanobacter*-related bacteria (ORB) strains and their closest reference sequences. Bootstrap values (> 50%) based on 1,000 resamplings are given at the nodes. The labels in blue and black bold font indicate the sequences obtained from the strains isolated in this study and the type strains of the ORB, respectively. Reference sequences in different shades of color indicate that they originated from different bacterial orders: red, *Oceanospirillales*; purple, *Pseudomonadales*; green, *Burkholderiales*.

Baeyer-Villiger monooxygenase (BVMO) belongs to a flavin-binding monooxygenase superfamily that catalyzes the insertion of an oxygen atom in a C–C bond using dioxygen and NADPH (Fraaije et al., 2002). Previous studies have shown that BVMOs are involved in long-chain alkane degradation in some species of the genera *Thalassolituus* (Gregson et al., 2018), *Acinetobacter* (Minerdi et al., 2012), and *Alcanivorax* (Liu et al., 2011). In this study, BVMO-like proteins were found in both HD and NHD members. The BVMO homologs from HD members all show very high amino acid sequence identities (61.3–100%) to the BVMO (CCU71147) of strain MIL-1^T^ (Supplementary Table 5). In contrast, the BVMOs from NHD members show only 22.5–24.4% identities, indicating that they most likely were not active in hydrocarbon degradation, consistent with the phenotype of NHD members being unable to degrade hydrocarbons. The BVMO tree (Figure 8) also had a similar topology to the genomic phylogenetic tree (Figure 2A). In detail, the BVMOs from each clade of the HD group were clustered together, and their closest homologs were mainly derived from the orders *Oceanospirillales* and *Pseudomonadales*. Notably, although the BVMOs of NHD members clustered together, they were far from those of HD members.

In hydrocarbon-degrading bacteria, ferredoxin and ferredoxin reductase are known to transfer electrons to monooxygenases, such as AlkB and BVMO. In this study, the ferredoxins of the HD and NHD members separately have 48.3–100% and 24.1–37.9% amino acid sequence identities (Supplementary Table 5) to their counterpart CCU73057 in strain MIL-1^T^, implying that this protein is more conserved in HD than in NHD. In contrast, the ferredoxin reductases derived from both HD and NHD members all show very high identities (82.9–100%) to their counterpart (CCU72773), indicating that they are highly conserved in ORB bacteria.

Alcohol and aldehyde dehydrogenases sequentially oxidize the alkanols to the corresponding aldehydes and fatty acids, respectively. The two dehydrogenases are widely present in HD and NHD members with various identities to their counterparts (CCU73168 and CCU70671 in strain MIL-1^T^) (Supplementary Table 5). Notably, some alcohol dehydrogenases are absent in the members of the S7-S9 clades within the HD group, which all originated from the Pacific Ocean with the exception of MAG UBA2009 (Supplementary Table 2), indicating that these Pacific Ocean-originated bacteria perhaps recruit other alcohol dehydrogenases to oxidize the alkanols. Further genomic analysis also demonstrated that these bacteria all contained various types of alcohol dehydrogenases (data not shown).

A previous study showed that an esterase (CCU71342) was required to hydrolyze the ester produced by BVMO to generate an alcohol and a fatty acid in strain *T. oleivorans* MIL-1^T^ (Gregson et al., 2018). Esterases closely related to CCU71342 are present in both HD and NHD members. Similar to the BVMOs, the esterases retrieved from HD members had obviously higher amino acid sequence identities (65.6 ± 19.2%) with the esterase CCU71342 than those from NHD members (26.8 ± 0.7%; Supplementary Table 5), implying that the esterases of NHD members, like their BVMOs, may also not be active in alkane degradation.

### Phenotypic characteristics of two novel species

The two strains, alknpb1M-1^T^ and 59MF3M-4^T^, were characterized as Gram-stain negative, motile, and strictly aerobic. Their cells are curved rod-shaped, 0.2–0.4 μm wide and 1.4–4.4 μm long and contain a single polar flagellum (Supplementary Figure 7). Their colonies were observed to be white, smooth, circular, opaque with entire margins and 1–2 mm in diameter on a modified MA plate at 25°C. Growth of the two strains was observed at 4–45°C with an optimum of 25–28°C and at pH 6.0–10.0 with an optimum of pH 7.0–8.0. They can tolerate 8% (w/v) NaCl and grow well under 3%. For enzyme activity and biochemical characteristics, the two strains and the three reference type strains were analyzed in parallel using the API ZYM, 20NE, and 20E strips. Generally, they showed similar physiological characteristics; both strains alknpb1M-1^T^ and 59MF3M-4^T^ could hydrolyze gelatin and showed positive activities of gelatinase and α-glucosidase. Hydrocarbon-utilizing test showed that strain alknpb1M-1^T^ could actively grow with middle chain-length linear alkanes (C_15_H_32_–C_24_H_50_). However, the results of two independent repeated experiments showed that strain 59MF3M-4^T^ could not utilize any of the tested alkanes as sole carbon and energy sources (Supplementary Figure 8). Given the presence of the alkane degradative pathway in the genome, this presumably reflects failure to activate alkane utilization under our experimental conditions. The major fatty acids in the two strains (> 10%) were identified as C_16:1_ ω7/6*c*, C_16:0_, and C_18:1_ ω7*c*, in line with the fatty acid profiles of other reported *Thalassolituus* species (Table 2 and Supplementary Table 6). Although the fatty acid profiles from these strains were similar, their proportions were different from each other. The predominant respiratory quinone of the two strains was found to be Q-9, which is consistent with the quinone profiles of the known *Thalassolituus* and *Oleibacter* species. The polar lipids of the two strains were found to include phosphatidylethanolamine, phosphatidylglycerol, diphosphatidylglycerol, aminolipid, glycolipid, and several unidentified phospholipids and polar lipids (Supplementary Figure 9). Notably, glycolipids were detected only in strain 59MF3M-4^T^. Other differences in phenotypic, physiological, and chemotaxonomic characteristics among the two strains and their closely related species are shown in Table 2 and in the species descriptions.

**TABLE 2:** Differentiating characteristics of the type strains of the type species within the *Oceanobacter*-related bacteria.

Characteristic	1	2	3	4	5	6	7	8
**Isolation source**	Deep-sea bottom water	Unidentified sponge on deep-sea bottom	Coastal mixed seawater/sediment	Coastal surface seawater	Deep-sea seawater of the Mariana Trench	Coastal surface seawater	Ocean surface seawater	Coastal mangrove sediment
**Morphology**								
Cell shape	curved rods	curved rods	curved rods	curved rods	curved rods	curved rods	straight rods	rod
Flagella	single polar flagellum	single polar flagellum	single or multiple polar flagella	single polar flagella	single polar flagella	single polar flagella	single polar flagella	single polar flagellum
Motility	+	+	+	+	+	+	+	+
Clone color	white	creamy white	creamy white	beige	light beige	nd	nd	milky white
**Temperature for growth (°*C*)**								
Range	4–35	10–45	4–30	15–42	4–37	10–40	10–40	20–40
Optimum	25	28	20–25	25	37	25–30	25–30	35
**pH for growth**								
Range	5–10	6–8	7.5–9	6–9	6–9	6–10	5–9	6–8
Optimum	7–8	6–7	8	7–8	6–7	6–9	nd	7–8
**NaCl for growth (w/v,%)**								
Range	1–7	1–8	0.5–5.7	0.5–5	0–8	1–7	1–7	0–5
Optimum	3	3	2.3	2.5	5%	nd	nd	0.5
**Reduction of nitrate**	+	+	−	+	+	+	+	+
**Voges–Proskauer**	−	−	+	−	−	−	−	−
**Hydrolysis of**								
Starch	−	−	−	+	−	nd	nd	−
Gelatin	+	+	−	−	−	+	−	−
**Enzyme activities**								
Arginine dihydrolase	−	−	−	−	−	−	+	−
Urease	+	+	+	+	+	+	−	−
lipase (C14)	+	+	+	+	+	+	−	+
cystine aminopeptidase	+	w	+	+	w	+	−	w
α-glucosidase	+	w	−	−	+	−	−	nd/-
Arginine dihydrolase	−	−	−	−	−	−	+	−
Gelatinase	+	+	−	−	−	−	−	−
**Utilization**								
*D*-glucose	−	−	−	−	−	−	+	+
Trisodium citrate/Citrate	−	−	−	−	−	−	+	+
**Acid production**								
Glucose	−	−	−	−	−	−	+	+
**Major fatty acids (> 10%)**	C16:1 ω7/6c; C16:0; C18:1 ω7c/6c	C16:1 ω7/6c; C16:0; C18:1 ω7c/6c	C16:1 ω7/6c; C14:0; C16:0	C16:1 ω7/6c; C18:1 ω7c; C16:0	C16:1 ω7/6c; C16:0; C18:1 ω7c/6c	C16:1 ω7c; C16:0; C18:1 ω6/ω9c	C18:1 ω7c; C16:0; C16:1 ω7c	C16:0; C18:1 ω7/6c; C18:0; C16:1 ω7/6c
**Isoprenoid quinone**	Q-9 (96.1%) with minor Q-8 (3.9%)	Q-9	Q-9 (97%) with minor Q-8 (3%)	Q-9	Q-9	Q-9 (97–99%) with minor Q-8 (1–3%)	Q-8 (91%) with minor Q-7 (7%), Q-9 (2%)	Q-8
**Major polar lipids**	PE, PG, DPG, AL, PL, L	PE, PG, DPG, AL, GL, L	PE, PG, DPG, AL	PE, PG, DPG, AL	PE, PG, DPG, AL	NP, PG, GL	PG, NP, GL	PG, PE, AL, L
**alkB gene**	+	+	+	+	+	+	−	−
**DNA G + C content (%)#**	53.4	52.9	46.6	53.1	53.1	51.4	55.3	54.3

Strains: 1, alknpb1M-1^T^; 2, 59MF3M-4^T^; 3, *T. oleivorans* MIL-1^T^; 4, *T. marinus* IMCC1826^T^; 5, *T. alkanivorans* TMPB967^T^; 6, *Oleibacter marinus* DSM 24913^T^; 7, *Oceanobacter kriegii* IFO 15467^T^; 8, *Oceanobacter mangrovi* SM2-42^T^. Except for strains 6–8, the data of API 20NE, API 20E, and API ZYM for all other type strains were examined in this study. #: data retrieved from genome sequences. nd, no data available; + , present/tested positive; –, absent/tested negative; w, weakly positive; PE, phosphatidylethanolamine; PG, phosphatidylglycerol; DPG, diphosphatidylglycerol; AL, unidentified aminolipid; PL, unidentified phospholipid; GL, unidentified glycolipid; L, unknown polar lipid; NP, ninhydrin-positive phospholipid.

### Distinction in phenotypic characteristics between HD and NHD members

In addition to the differences in genome-based phylogenies, relatedness indices and comparative genomic analysis, phenotypic, genotypic and chemotaxonomic characteristics are also important indicators of genus and species delineation. Based on our results and previous reports, at least three characteristics could readily distinguish the members of the HD group (the expanded genus *Thalassolituus* in this study) from the NHD group (Table 2). First, all HD members possess *alkB* genes and exhibit high alkane-degrading activities, the above results for strain 59MF3M-4^T^ excepting, whereas NHD members from the genus *Oceanobacter* cannot utilize alkanes and harbor no *alkB* genes. Second, the predominant respiratory quinone of all HD members is Q-9, whereas that of NHD members is Q-8. Thirdly, none of the HD bacteria can utilize *D*-glucose and trisodium citrate, but all NHD members can.

## Conclusion

In this study, four ORB-related strains were isolated from four distinct marine habitats in three ocean regions. Based on their genomes, together with the current available genomes of ORB, the taxonomic affiliation of ORB was clarified. Phylogenomic analysis demonstrated that bacteria of the genera *Thalassolituus* and *Oleibacter* within the hydrocarbon-degrading group formed a monophyletic branch and could be classified as the same genus. This distinctiveness was also supported by the genomic relatedness, comparative genomics analysis, phenotypic and chemotaxonomic characteristics. Therefore, we propose the combination of the two hydrocarbon-degrading genera by transfer of *Oleibacter marinus* into the genus *Thalassolituus*. However, due to homonymy with the prior name *Thalassolituus marinus* Choi and Cho, 2013, the species epithet in the name *O. marinus* must be replaced with a *nomen novum* in accordance with Rule 41a of the International Code of Nomenclature of Prokaryotes (Parker et al., 2019). Additionally, the dDDH and ANI values and several phenotypic characteristics support the conclusion that the newly isolated strains, alknpb1M-1^T^ and 59MF3M-4^T^, represent two novel species in the genus *Thalassolituus* which are named and described below. Overall, these findings build a phylogenetic framework for *Oceanobacter*-related bacteria and contribute to understanding of their role in marine hydrocarbon and carbon cycles.

## Taxonomic proposals

### Emended description of the genus *Thalassolituus*

The description of the genus *Thalassolituus* is as given in Yakimov et al. (2004) with the following amendment: species are variable for the reduction of nitrate, gelatinase activity, and abilities of hydrolyzing starch and gelatin. The principal fatty acids (> 10%) are C_16:0_, C_16:1_ ω7/6c, C_18:1_ ω7/6*c*, and C_18:1_ ω6/ω9*c*. The major polar lipids are phosphatidylethanolamine, phosphatidylglycerol, diphosphatidylglycerol and unidentified aminolipids and glycolipids. The genome size range is 3.86–4.37 Mb with DNA G + C content of 46.6–53.4%. Although all species possess alkane monooxygenase encoding genes (*alkB*), some of them cannot utilize linear alkanes as the sole carbon and energy source for growth. The type species of the genus is *Thalassolituus oleivorans*.

### Description of *Thalassolituus hydrocarbonoclasticus* sp. nov.

*Thalassolituus hydrocarbonoclasticus* (hy.dro.car.bo.no. clas’ti.cus N.L. neut. n. *hydrocarbonum*, hydrocarbon; N.L. adj. *clasticus*, breaking; from Gr. adj. *klastos* -ê -on, broken in pieces; N.L. masc. adj. *hydrocarbonoclasticus*, hydrocarbonoclastic, breaking hydrocarbon).

Cells are Gram-stain negative, strictly aerobic, curved rods, 0.3–0.4 μm in width and 2.4–4.4 μm in length, motile by means of a single polar flagellum. Colonies are white, smooth, circular, opaque with entire margins and 1–2 mm in diameter after growth on modified MA supplemented with 1 g/L sodium acetate at 25°C for 3 days. Growth occurs at 4–35°C (optimum 25°C), at pH 5–10 (pH 7–8) and in the presence of 1–7% NaCl (w/v) (3%). Positive for catalase and oxidase. Hydrolyzes Tween 80 and gelatin, but not starch or aesculin. In the API ZYM tests, cells are positive for the activities of alkaline phosphatase, esterase (C4), esterase lipase (C8), lipase (C14), leucine aminopeptidase, valine aminopeptidase, cystine aminopeptidase, acid phosphatase, naphthol-AS-Bl-phosphoamidase, and α-glucosidase, but are negative for all other tests. In the API 20NE tests, cells are positive for nitrate reduction, urease, and gelatin hydrolysis, but negative for all other tests. In the API 20E tests, cells are only positive for urease and gelatinase. Able to grow with middle chain-length linear alkanes (C_15_H_32_–C_24_H_50_) as sole carbob source. The principal fatty acids (> 5%) are C_16:1_ ω7/6*c*, C_16:0_, and C_18:1_ ω7*c*. The predominant respiratory quinone is Q-9, with a minor amount of Q-8. The polar lipids are phosphatidylethanolamine, phosphatidylglycerol, diphosphatidylglycerol, and several unidentified aminolipids, phospholipids and lipids.

The type strain, alknpb1M-1^T^ (= MCCC 1A17715^T^ = KCTC 82141^T^) was isolated from deep-sea sediment overlying water of the South China Sea. The type strain genome size is 4,067,951 bp with a DNA G + C content of 53.4% (Genbank accession number CP054475).

### Description of *Thalassolituus pacificus* sp. nov.

*Thalassolituus pacificus* (pa.ci’fi.cus. L. masc. adj. *pacifica*, peaceful, pertaining to the Pacific Ocean).

Cells are Gram-stain negative, strictly aerobic, curved rods, 0.2–0.3 μm in width and 1.4–2.3 μm in length, motile by means of a single polar flagellum. Colonies are creamy white, smooth, circular, opaque with entire margins and 1–2 mm in diameter after growth on modified MA supplemented with 1 g/L sodium acetate after at 25°C for 3 days. Growth occurs at 10–45°C (optimum 28°C), at pH 6–8 (pH 7) and in the presence of 1–8% NaCl (w/v) (3%). Positive for catalase and oxidase. Hydrolyzes Tween 80 and gelatin, but not aesculin and starch. In the API ZYM tests, cells are positive for the activities of alkaline phosphatase, esterase (C4), esterase lipase (C8), lipase (C14), leucine aminopeptidase, valine aminopeptidase, cystine aminopeptidase, acid phosphatase and naphthol-AS-Bl-phosphoamidase; weakly positive for α-glucosidase; and negative for all other tests. In the API 20NE tests, cells are positive for nitrate reduction, urease, and gelatin hydrolysis, but negative for all other tests. In the API 20E tests, cells are only positive for urease and gelatinase. The principal fatty acids (> 5%) are C_16:1_ ω7/6*c*, C_16:0_, and C_18:1_ ω7*c*. The respiratory quinone is Q-9. The polar lipids are phosphatidylethanolamine, phosphatidylglycerol, diphosphatidylglycerol, and several unidentified aminolipids, glycolipids, and lipids.

The type strain, 59MF3M-4^T^ (= MCCC M21136^T^ = KCTC 92589^T^) was isolated from an unidentified deep-sea sponge in the Western Pacific Ocean. The type strain genome size is 4,266,791 bp with a DNA G + C content of 52.9% (Genbank accession number JAOANI000000000).

### Description of *Thalassolituus maritimus* nom. nov.

*Thalassolituus maritimus* (ma.ri’ti.mus. L. masc. adj. *maritimus*, of the sea, marine, maritime).

Basonym: *Oleibacter marinus* Teramoto et al., 2011.

The description of *Thalassolituus maritimus* is identical to that given for *Oleibacter marinus* (Teramoto et al., 2011).

The type strain is 2O1^T^ (= NBRC 105760^T^ = BTCC B-675^T^ = DSM 24913^T^).

## Data availability statement

The datasets presented in this study can be found in online repositories. The names of the repository/repositories and accession number(s) can be found in the article/Supplementary material.

## Author contributions

ZS supervised the study. CD and ZS designed the research outline. CD, LW, and JW performed the experiments. CD analyzed the data and drafted the manuscript. ZS, QL, and ZH revised the manuscript. All authors read and approved the final manuscript.

## Footnotes

1
https://www.ezbiocloud.net/

2
https://tygs.dsmz.de/
