# Supplementary material for: Genome-based taxonomic rearrangement of Oceanobacter-related bacteria including the description of Thalassolituus hydrocarbonoclasticus sp. nov. and Thalassolituus pacificus sp. nov. and emended description of the genus Thalassolituus
Source: Front Microbiol. 2022 Dec 20;13:1051202. doi: 10.3389/fmicb.2022.1051202 (PMC9807766; doi:10.3389/fmicb.2022.1051202)
Supplement: Supplementary file 1 [file Data_Sheet_1.docx]

Supplementary Figures


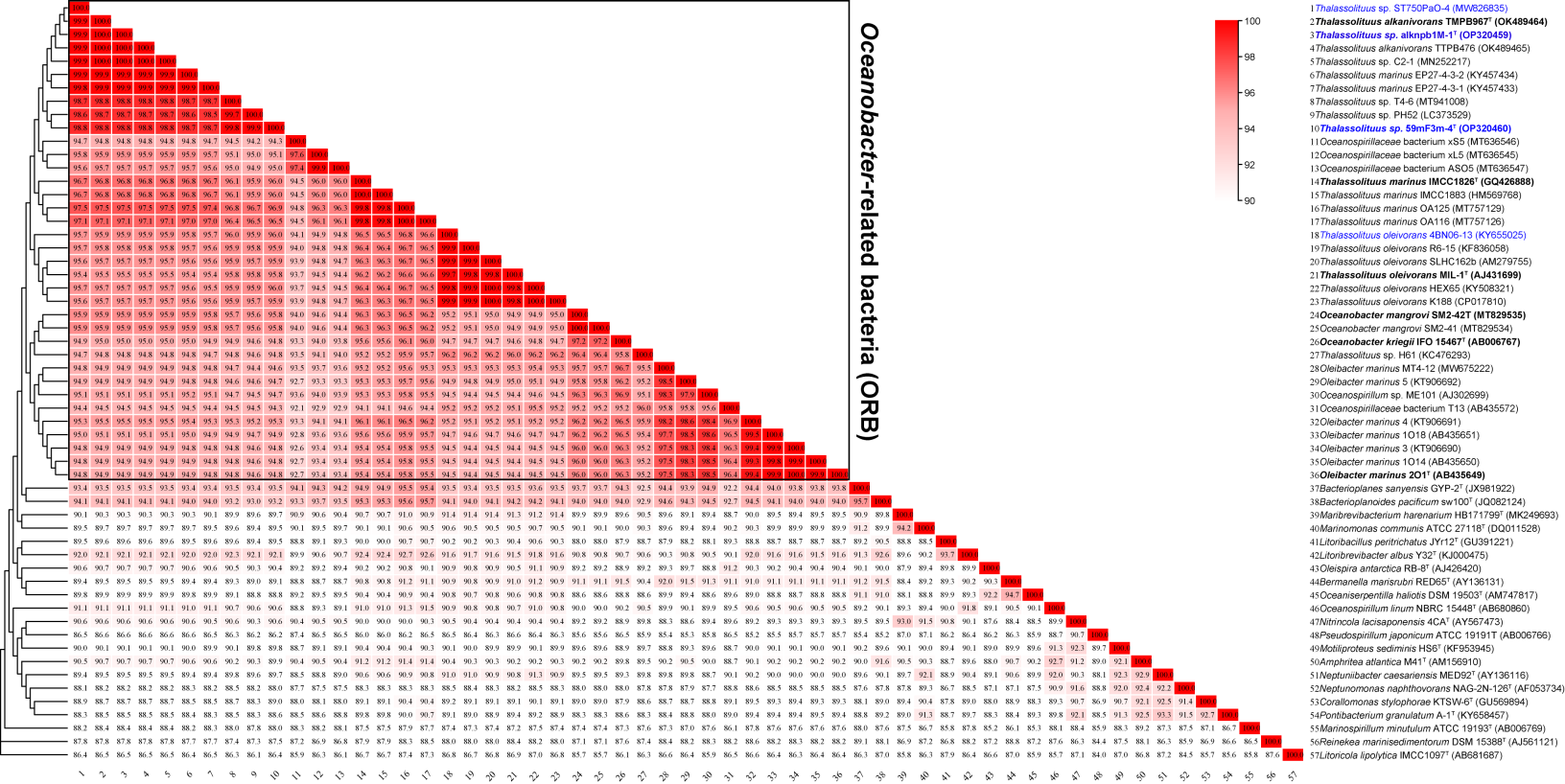


**Supplementary Figure 1.** 16S rRNA gene sequence pairwise similarity percentages among the *Oceanobacter*-related bacteria (ORB) and other type strains of the type species within the family *Oceanospirillaceae*. The pairwise 16S rRNA gene sequence similarities were calculated based on 1,448 nucleotide positions. The boxed region contains all currently known strains belonging to the ORB. Strains isolated in this study and the type strains of ORB are highlighted in blue and black bold, respectively.

­­
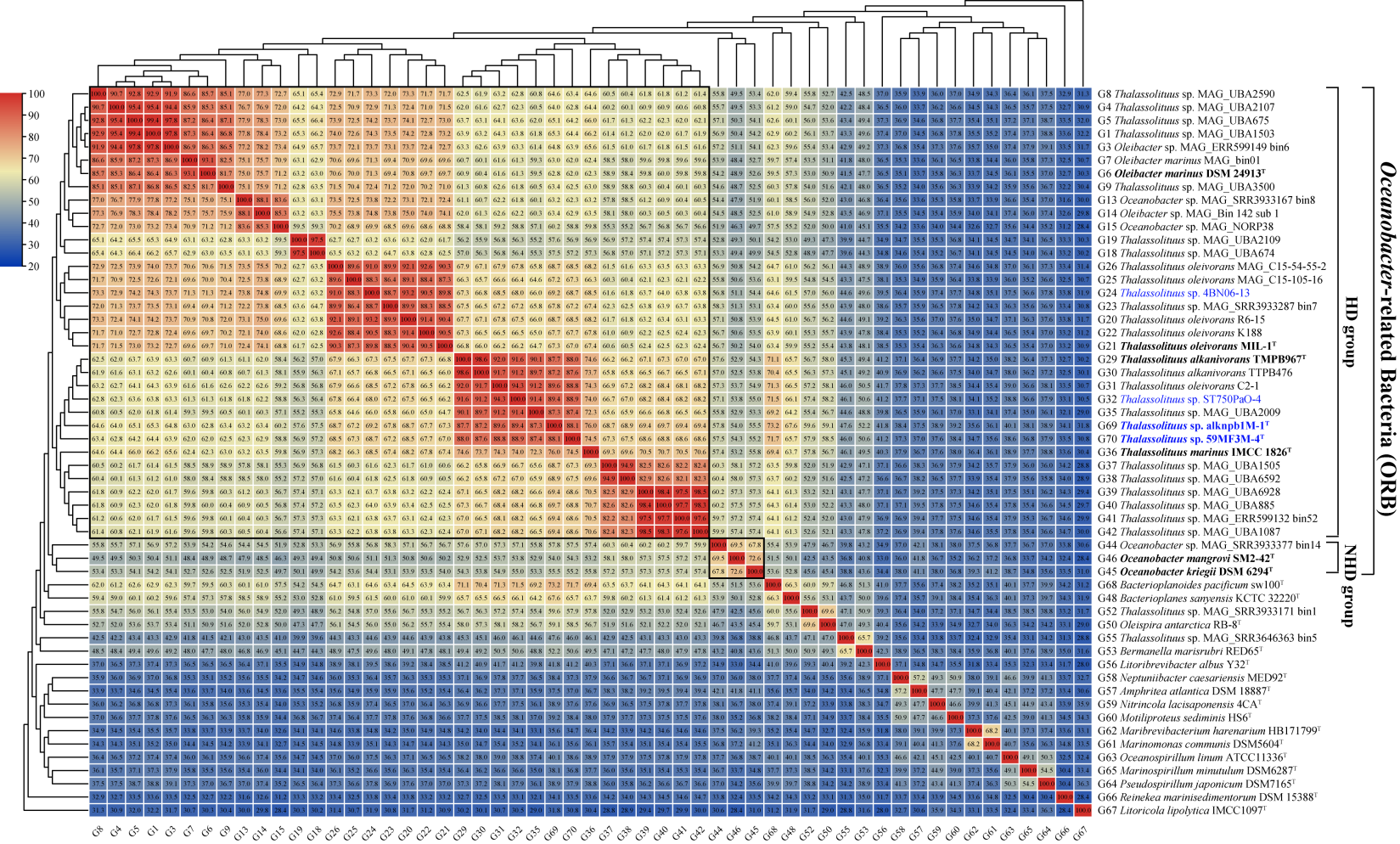


**Supplementary Figure 2.** POCP value matrices from pairwise genome comparisons. The heatmap shows POCP values (in each color block) between genomes, along with the phylogenomics tree (Figure 2A) cladogram to show relationships. The boxed regions indicate inferred genera within the ORB based on POCP comparisons, as well as monophyly in the genome-based phylogenetic tree (Figure 2A). Similarly, inferred genera were also observed from the AAI value matrices (Figure 3). Genomes retrieved from the strains isolated in this study and the type strains of ORB are highlighted in blue and black bold, respectively. Genomes retrieved from the metagenome-assembled genomes are marked by "MAG" in their names.


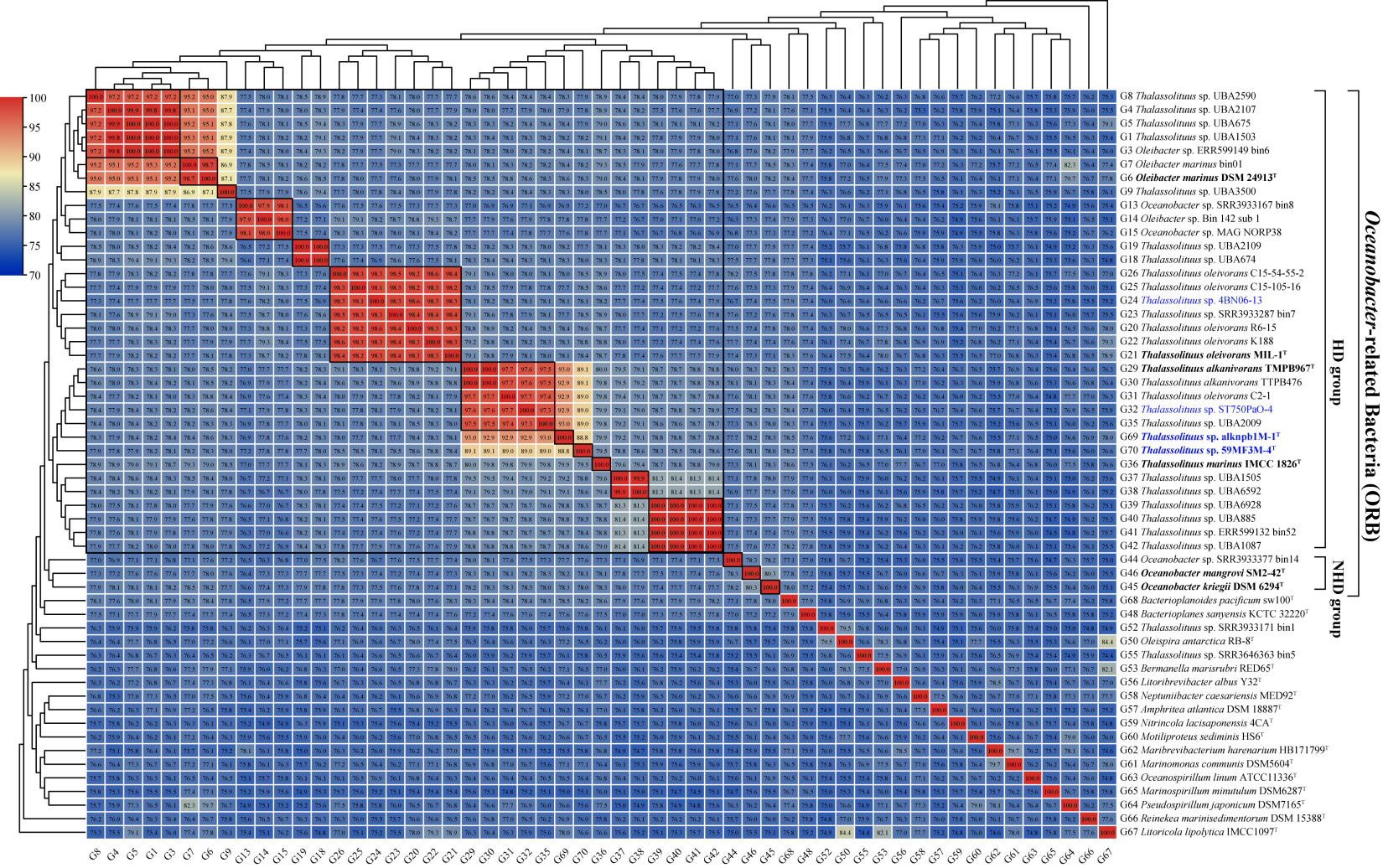


**Supplementary Figure 3.** ANI value matrices from pairwise genome comparisons. The heatmap shows ANI values (in each color block) between genomes, along with the phylogenomics tree (Figure 2A) cladogram to show relationships. The largest boxed region indicates the newly revised genus *Thalassolituus*. Twelve small boxed regions indicate the inferred species clusters within the newly revised genus *Thalassolituus* based on ANI comparisons, as well as monophyly in the genome-based phylogenetic tree (Figure 2A). Identically inferred species clusters were also observed from the dDDH value matrices (Figure 4). Genomes retrieved from the strains isolated in this study and the type strains of ORB are highlighted in blue and black bold, respectively. Genomes retrieved from the metagenome-assembled genomes are marked by "MAG" in their names.


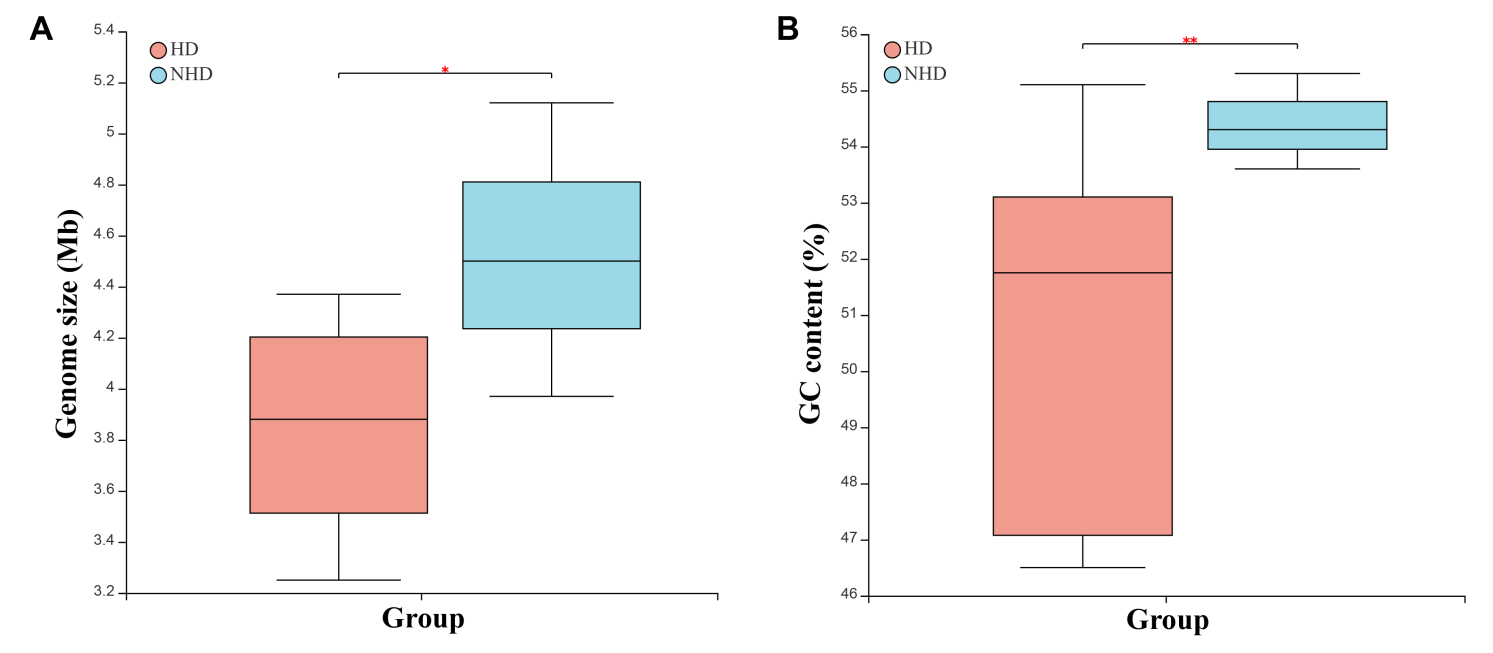


**Supplementary Figure 4.** Variations in genome size and G+C content between the HD and NHD groups in ORB. The significant level in variations was determined using the Wilcoxon test (*, *P*< 0.05; **, *P*< 0.01).


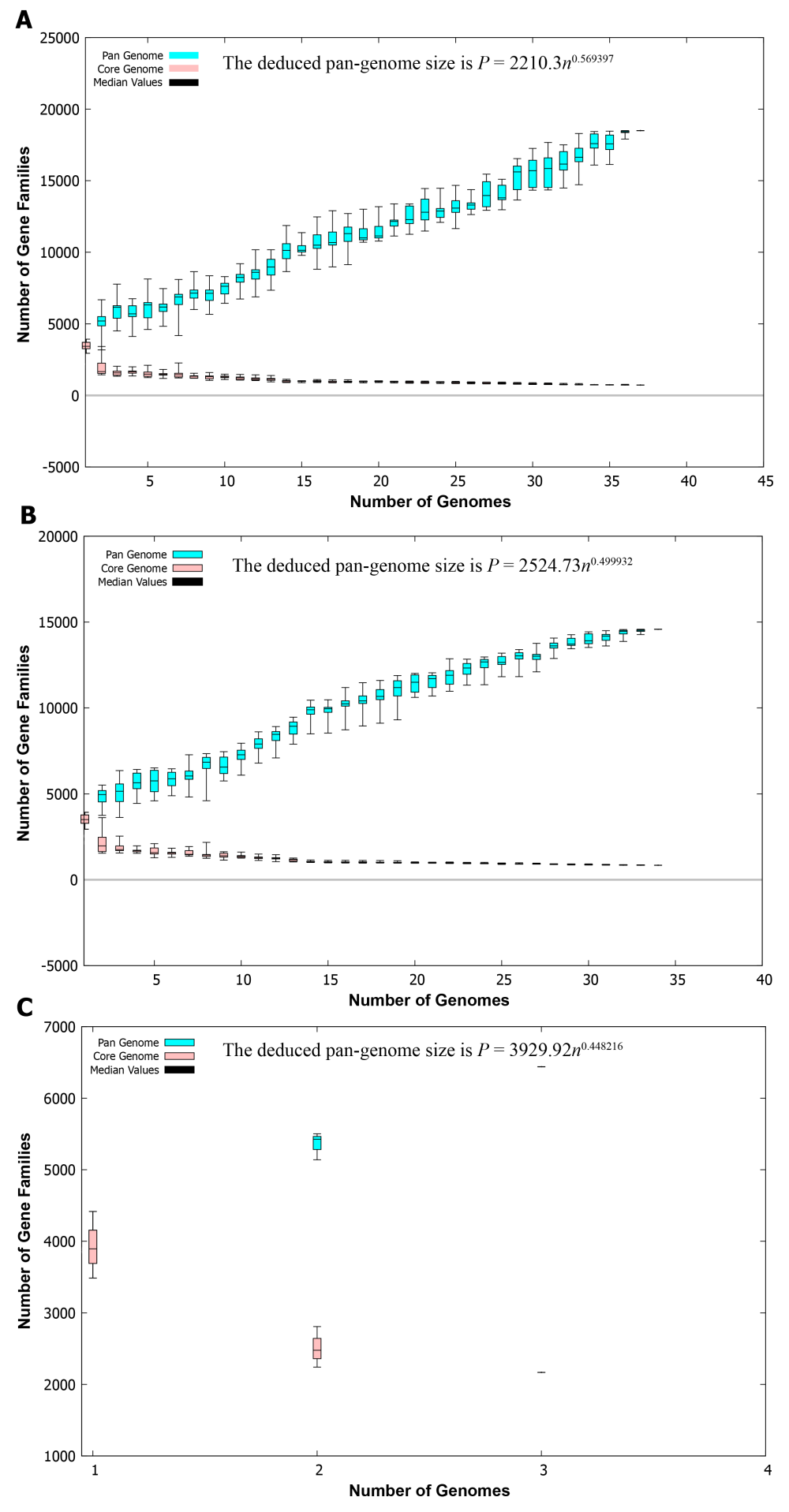


**Supplementary Figure 5.** The pangenome curves of ORB strains. (A) All ORB strains; (B) HD strains; (C) NHD strains.


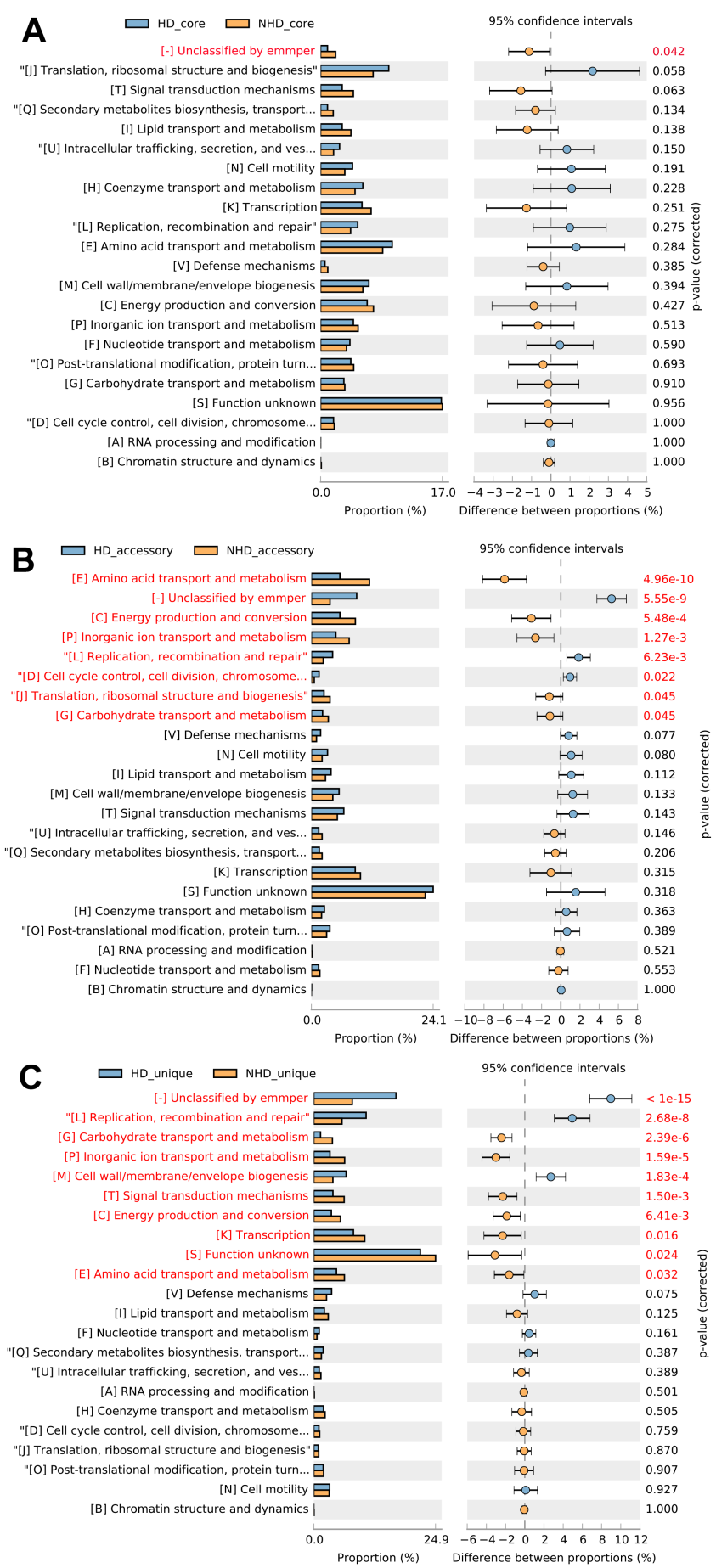


**Supplementary Figure 6.** Distributions of COG categories between HD and NHD bacteria. (A) COG categories of core genes. (B) COG categories of accessory genes. (C) COG categories of unique genes. Blue and orange bars represent HD and NHD bacteria, respectively. The COG category marked in red indicates that its proportion is significantly different (*p* <0.05) between the HD and NHD groups.

**
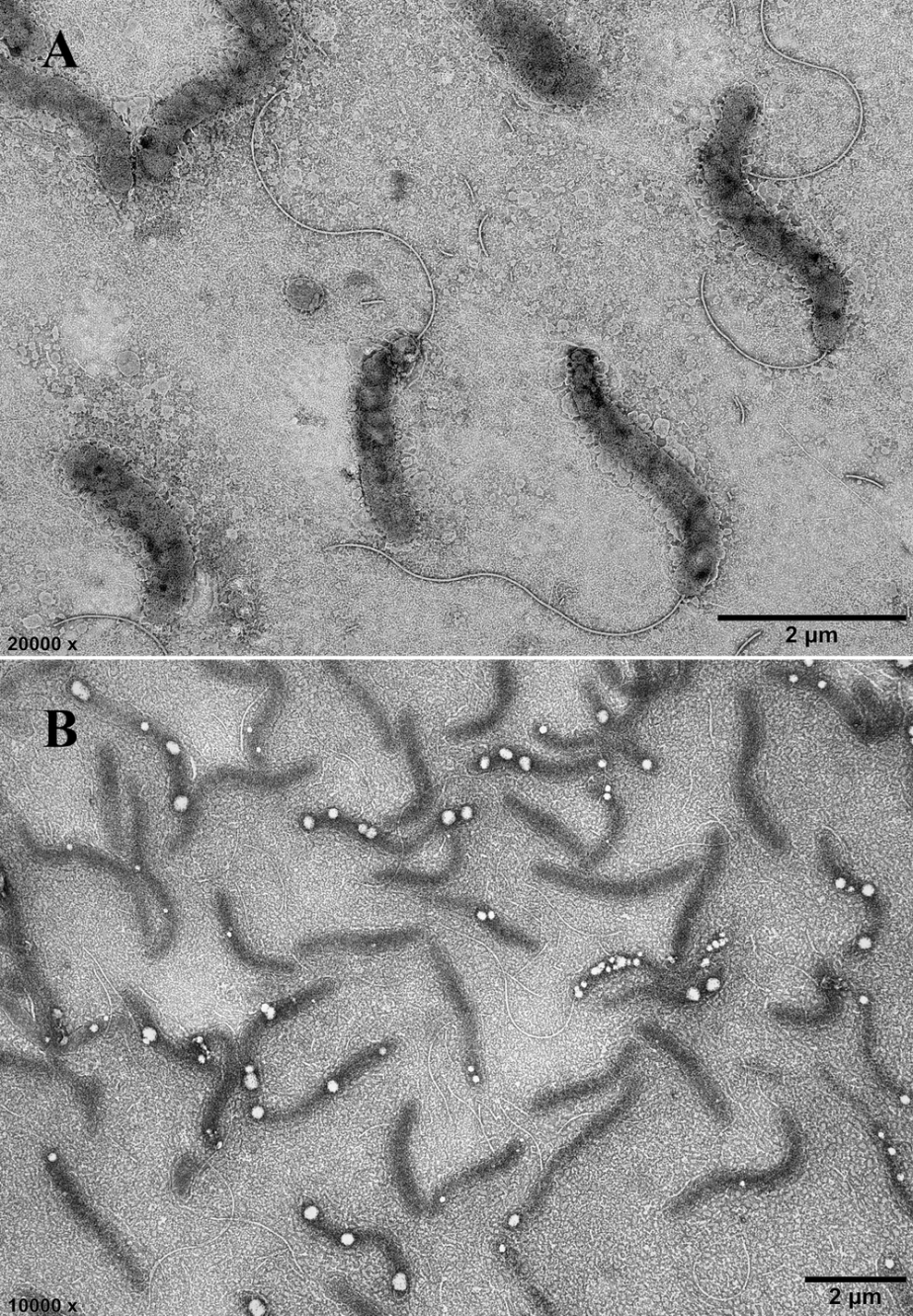
**

**Supplementary Figure 7.** Transmission electron micrograph of negatively stained cells of strains alknpb1M-1^T^ (A) and 59MF3M-4^T^ (B) grown on modified marine agar 2216 (MA, BD Difco^TM^) supplemented with 1 g L^-1^ sodium acetate for 48 h at 25 °C.


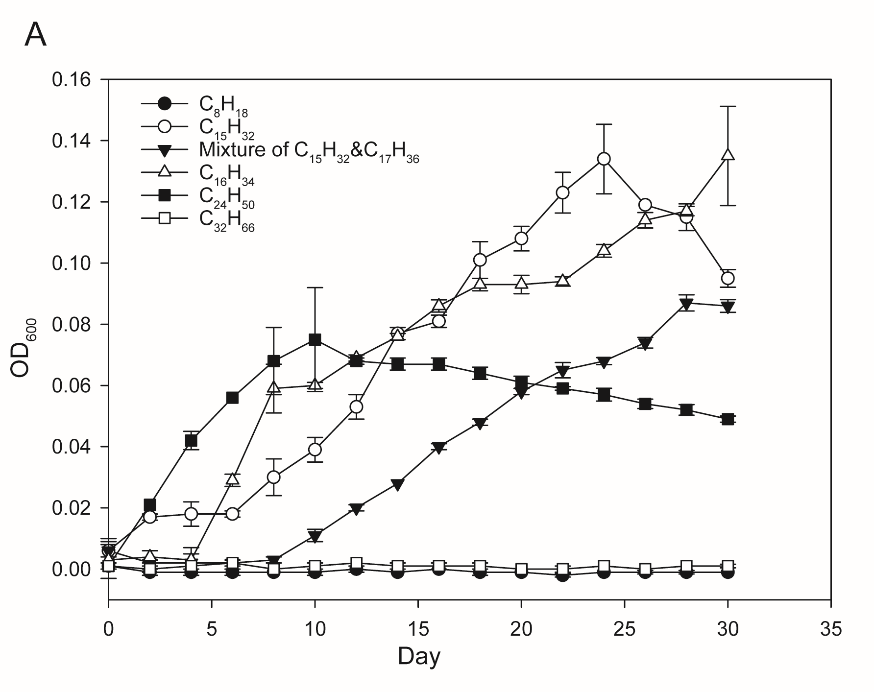


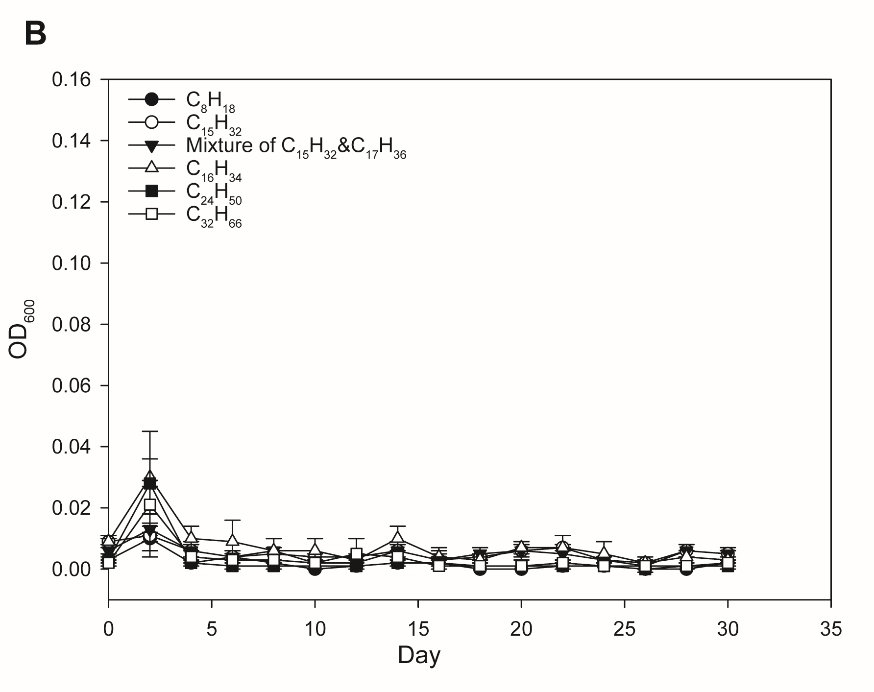


**Supplementary Figure 8.** Growth curves of strains alknpb1M-1^T^ (A) and 59MF3M-4^T^ (B) using various chain-length linear alkanes as sole carbon and energy sources in ONR7a medium at 25 °C.

**
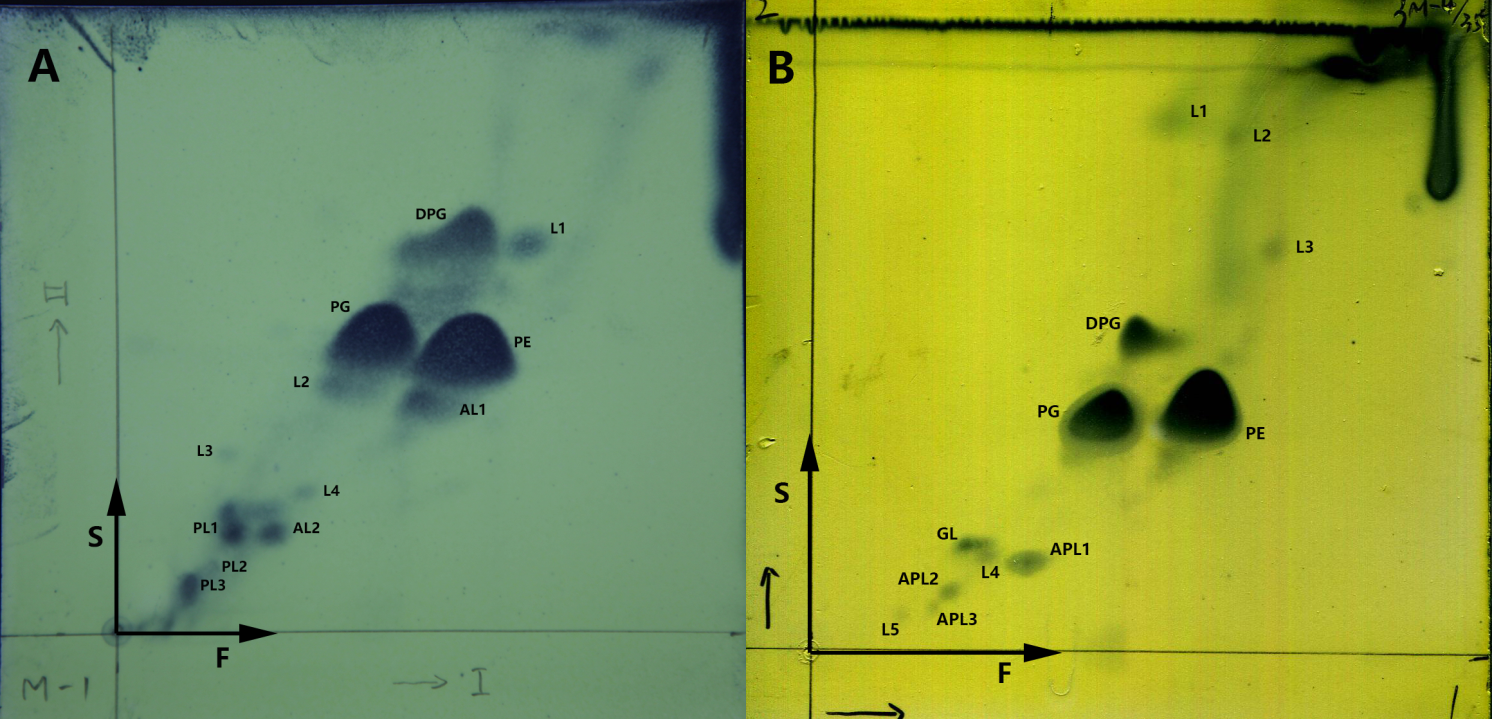
**

**Supplementary Figure 9.** The polar lipid profiles of strains alknpb1M-1^T^ (A) and 59MF3M-4^T^ (B) were identified as seven kinds of major components: PE, phosphatidylethanolamine; PG, phosphatidylglycerol; DPG, diphosphatidylglycerol; AL, unidentified aminolipid; PL, unidentified phospholipid; GL, glycolipid; and L, unknown polar lipid. F, first dimension of TLC; S, second dimension of TLC. The TLC plates shown here were stained with molybdatophosphoric acid.
